# *Borrelia burgdorferi* Requires the Alternative Sigma Factor RpoS for Dissemination within the Vector during Tick-to-Mammal Transmission

**DOI:** 10.1371/journal.ppat.1002532

**Published:** 2012-02-16

**Authors:** Star M. Dunham-Ems, Melissa J. Caimano, Christian H. Eggers, Justin D. Radolf

**Affiliations:** 1 Department of Medicine, University of Connecticut Health Center, Farmington, Connecticut, United States of America; 2 Department of Biomedical Sciences, Quinnipiac University, Hamden, Connecticut, United States of America; 3 Department of Pediatrics, University of Connecticut Health Center, Farmington, Connecticut, United States of America; 4 Department of Genetics and Developmental Biology, University of Connecticut Health Center, Farmington, Connecticut, United States of America; 5 Department of Immunology, University of Connecticut Health Center, Farmington, Connecticut, United States of America; Medical College of Wisconsin, United States of America

## Abstract

While the roles of *rpoS_Bb_* and RpoS-dependent genes have been studied extensively within the mammal, the contribution of the RpoS regulon to the tick-phase of the *Borrelia burgdorferi* enzootic cycle has not been examined. Herein, we demonstrate that RpoS-dependent gene expression is prerequisite for the transmission of spirochetes by feeding nymphs. RpoS-deficient organisms are confined to the midgut lumen where they transform into an unusual morphotype (round bodies) during the later stages of the blood meal. We show that round body formation is rapidly reversible, and *in vitro* appears to be attributable, in part, to reduced levels of Coenzyme A disulfide reductase, which among other functions, provides NAD^+^ for glycolysis. Our data suggest that spirochetes default to an RpoS-independent program for round body formation upon sensing that the energetics for transmission are unfavorable.

## Author Summary

Lyme disease, caused by the spirochetal pathogen *Borrelia burgdorferi*, is the most prevalent arthropod-borne infection in the United States. In order to maintain itself in nature, *B. burgdorferi* must cycle between its arthropod vector, *Ixodes* ticks, and a mammalian reservoir, usually a small rodent. Previous studies have demonstrated that the alternative sigma factor RpoS is essential for *B. burgdorferi* to infect a mammalian host, whereas a role within the tick has never been examined. In this study, we determined that one or more RpoS-dependent genes are required for *B. burgdorferi* to disseminate through the tick. Using a combination of microscopy techniques, we show that RpoS-deficient organisms are confined to the lumen of the tick midgut during nymphal feeding where they form round bodies, while wild-type spirochetes remain elongated and traverse the midgut to enter the hemolymph and salivary glands *en route* to the mammalian host.

## Introduction

Bacterial genomes typically encode multiple alternative sigma factors that reversibly associate with the RNA polymerase apoenzyme to promote the transcription of specific subsets of genes in response to changing environmental conditions [1]. One of the best studied alternative sigma factors is RpoS, the master regulator of the general stress response in *E. coli* (RpoS_E*c*_) [2], [3]. Regulation of *rpoS_Ec_* is multifaceted, occurring at the transcriptional and translational levels, as well by proteolysis [2], [3]. Induction of the RpoS_Ec_ regulon occurs in response to a variety of stressors, including low nutrient availability, high osmolarity, reactive oxygen intermediates and low pH [4]. Up to 10% of the genome may be regulated either directly or indirectly by RpoS_Ec_ with the composition of the regulon determined by the specific stressor [5]. RpoS orthologs exist in diverse Proteobacteria, including numerous pathogenic species, and have been shown to control genes involved in a wide range of adaptive processes [6]. For example, RpoS is essential for biofilm formation in *Pseudomonas aeruginosa* [7], growth and survival of *Legionella pneumophila* within phagolysosomes [8], and infectivity of mice by *Salmonella enterica* [9].

*B. burgdorferi* (*Bb*), the Lyme disease spirochete, uses just three sigma factors, σ^70^, RpoN, and RpoS, to modulate its transcriptome during its enzootic cycle. Transcription of *rpoS*_Bb_ is directly activated by the alternative sigma factor RpoN (BB0450) with the aid of two enhancer proteins: the response regulator Rrp2 (BB0763) and the DNA-binding protein and Fur orthologue, BosR (BB0647) [10]–[12]. Additional levels of control are provided by the small RNAs *hfq* (*bb0268*) and *dsrA* (*bb0577*) as well as the carbon storage regulator *csrA* (*bb0184*) [12]. Based upon microarray analysis of mammalian host-adapted spirochetes [13], it was proposed that RpoS functions as a gatekeeper for controlling the upregulation of mammalian host-phase genes (e.g., *ospC*) and the downregulation of tick-phase genes (e.g., *ospA*). By quantitative real time PCR (qRT-PCR), we established that *rpoS* is induced during the nymphal blood meal but is not expressed by spirochetes within replete larvae or unfed nymphs, thereby delineating the RpoS ON (fed nymph) and OFF (fed larvae and unfed nymph) states during the tick phase of the enzootic cycle [13], [14]. Expression patterns of RpoS-dependent genes in the tick, however, reflect more than a simple ON/OFF bipartite regulation. For example, OspC is induced within the midgut during nymphal feeding but not by all spirochetes [14]–[16]. In addition, OspA continues to be expressed at high levels in the midgut after engorgement and is “OFF” only after organisms have completely mammalian host-adapted [14], [16]–[18]. Within the mammal, RpoS-dependent genes are required to establish infection and presumably continue to be expressed until spirochetes are acquired by naïve larvae [12], [14], [19], [20].

While the roles of *rpoS_Bb_* and individual RpoS-dependent genes have been studied extensively within the mammal [21], the contribution of the RpoS regulon to the tick-phase of the enzootic cycle has yet to be examined. In this study, we defined the contours of RpoS-dependent gene expression within larval and nymphal life stages and demonstrate, for the first time, that RpoS is required for the transmission of Lyme disease spirochetes by feeding nymphs. Although RpoS-deficient spirochetes replicate at wild-type levels during the nymphal blood meal, mutant organisms display an aberrant morphology, previously referred to as round bodies or cysts [22]–[24], as feeding progresses. We show that round body formation is rapidly reversible, and *in vitro* appears to be attributable, in part, to reduced levels of Coenzyme A disulfide reductase, which among other functions, provides NAD^+^ for glycolysis. Our findings reveal a previously unsuspected role for RpoS_Bb_ in the physiological adaptations required for tick-to-mammal transmission as well as the existence of a proposed default survival mode (round body formation) triggered when spirochetes are unable to produce sufficient energy for dissemination within the nymph.

## Results

### Lyme disease spirochetes require RpoS-dependent genes other than *ospC* to traverse the nymphal midgut

We began by devising an experimental strategy to test our hypothesis that one or more genes within the RpoS regulon are required for the dissemination of spirochetes during nymphal feeding. While it has long been known that *ospC* is induced during nymphal feeding [15], there has been controversy as to whether this lipoprotein functions in the tick or the mammal [19], [25], [26]. The strategy we devised also enabled us to clarify the site of OspC function. Because spirochetes lacking either RpoS or OspC are avirulent by needle inoculation [26], [27], we used the immersion method [28] to introduce wild-type (WT, CE162), Δ*rpoS* (CE174), and Δ*ospC* (CE303) isolates into naïve larvae (Table 1); larvae infected by immersion feeding were allowed to molt into nymphs that then were fed on naïve mice. As described in Materials and Methods and summarized in Table 2, we cultured (i) hemolymph at 72 h post-placement to monitor penetration through the midgut; (ii) skin excised from the bite site at 24, 48 and 72 h post-repletion to assess penetration through the salivary glands and transmission; and (iii) distant tissues at 2 and 4 weeks post-repletion to assess dissemination within the mouse. WT spirochetes were isolated from hemolymph at 72 h post-placement, from the bite site at 48 and 72 h post-repletion and from multiple tissues at 2 and 4 weeks post-repletion. The Δ*rpoS* isolate was not recovered from either hemolymph or distal sites, while the Δ*rpoS*-complemented isolate (CE467) was recovered from both. In contrast to Δ*rpoS* organisms, Δ*ospC Bb* were recovered from hemolymph at 72 h post-placement and the bite site but only at 48 h post-repletion. Importantly, the time frames during which WT and Δ*ospC* organisms were recovered from hemolymph cultures were highly similar in each experiment (n = 3; data not shown), suggesting that the cultured samples contained comparable numbers of organisms. Given that RpoS is essential for the stress responses of many bacteria [2], [6], it is possible that spirochetes lacking this sigma factor were unable to survive within the midgut during feeding. qPCR and semi-solid plating, however, revealed virtually identical burdens of WT, Δ*rpoS*, and Δ*ospC* isolates in unfed and replete nymphs; furthermore, similar incubation times were required for the recovery of WT and Δ*rpoS* organisms by semi-solid plating (Figure S1; *p*≥0.05). Comparable results were obtained using fluorescent Δ*rpoS* mutant and complemented strains described in subsequent sections. Thus, *B. burgdorferi* requires one or more RpoS-dependent genes to traverse the midgut, while OspC functions exclusively within the mammal.

**Table 1:** *Borrelia burgdorferi* strains used in this study.

Strain	Description	Antibiotic Resistance^1^	Reference
**CE162**	Wild-type 297 clone	NA	[27]
**CE174**	CE162 Δ*rpoS*; non-fluorescent *rpoS* mutant	Erm	[27]
**CE303**	CE162 Δ*ospC*; non-fluorescent *ospC* mutant	Kan	This study
**CE467**	CE174+*rpoS*/pCE320; non-fluorescent RpoS-complement	Erm and Kan	[36]
**Bb914**	CE162 with *P_flaB_-gfp* in cp26; fluorescent wild-type	Gent	[30]
**Bb1058**	CE174 with *P_flaB_-gfp* in cp26; fluorescent *rpoS* mutant	Gent and Erm	This study
**SE186**	Bb1058+ *rpoS*/pCE320; fluorescent RpoS-complement	Gent, Erm, and Kan	This study
**CE56**	CE162*+P_flaB_-gfp/*pCE320; fluorescent *flaB* transcriptional reporter	Kan	[59]
**CE103**	CE162+*P_ospA_-gfp/*pCE320; fluorescent *ospA* transcriptional reporter	Kan	[36]
**CE309**	CE162 Δ*cdr*; non-fluorescent	Erm	[29]
**CE1655**	CE309+*cdr/*pCE323; non-fluorescent CoADR-complement	Erm and Kan	[29]

1 Antibiotic resistance determined by growing spirochetes in the presence of the following antibiotics: erythromycin (Erm, 0.06 µg/ml); kanamycin (Kan, 400 µg/ml); and/or gentamycin (Gent, 50 µg/ml).

**Table 2:** One or more RpoS-dependent gene products, independent of *ospC*, are required for spirochete's to penetrate into the hemolymph; see Figure S1 for spirochete burden analyses.

Strain	Description	Hemolymph^a^^,^ b ^,^ c	Bite Site^b^^,^ d			Distal Tissue Sites^b^^,^ e			
			24 h	48 h	72 h	Ear^f^	Joint	Bladder	Heart
**CE162**	WT	21/25	ND	ND	ND	7/7	6/7	7/7	6/7
**Bb914**	WT-*gfp*	35/37	0/24	8/20	13/20	25/25	25/25	25/25	24/25
**CE303**	Δ*ospC*	31/34	0/20	7/20	0/20	0/5	0/5	0/5	0/5
**CE174**	Δ*rpoS*	0/39	ND	ND	ND	0/7	0/7	0/7	0/7
**Bb1058**	Δ*rpoS*-*gfp*	0/56	0/24	0/24	0/24	0/25	0/25	0/25	0/25
**CE467**	Δ*rpoS+rpoS*	22/25	ND	ND	ND	7/7	7/7	7/7	7/7
**SE186**	Δ*rpoS*-*gfp+rpoS*	44/47	0/24	3/24	9/24	24/25	25/25	25/25	23/25

a Hemolymph was collected from feeding nymphs 72 h post-placement and cultured in BSK-II. The denominator represents the total number of ticks analyzed.

b Cultures were monitored for the presence of spirochetes by dark field microscopy for 8 weeks.

c The time frames in which organisms (WT, WT-*gfp*, *ΔospC*, *ΔrpoS*+*rpoS*, and *ΔrpoS-gfp*+*rpoS*) were recovered from the hemolymph was highly similar in each experiment (n = 3).

d Skin of a C3H/HeJ mouse (4–6 sites per mouse) was excised from the site where a nymph was attached at the indicated time post-repletion. A minimum of 3 mice were tested per isolate. The denominator represents the total number of bite sites analyzed; ND = not determined.

e Mice were sacrificed 4 weeks post-inoculation and the indicated tissues cultured in BSK-II. The denominator represents the total number of mice analyzed per isolate.

f Ear punches were performed at 2 and 4 weeks post-feeding and cultured in BSK-II.

### RpoS-dependent gene expression by spirochetes in fed nymphs differs from that of mammalian host-adapted organisms

**Figure 1:**
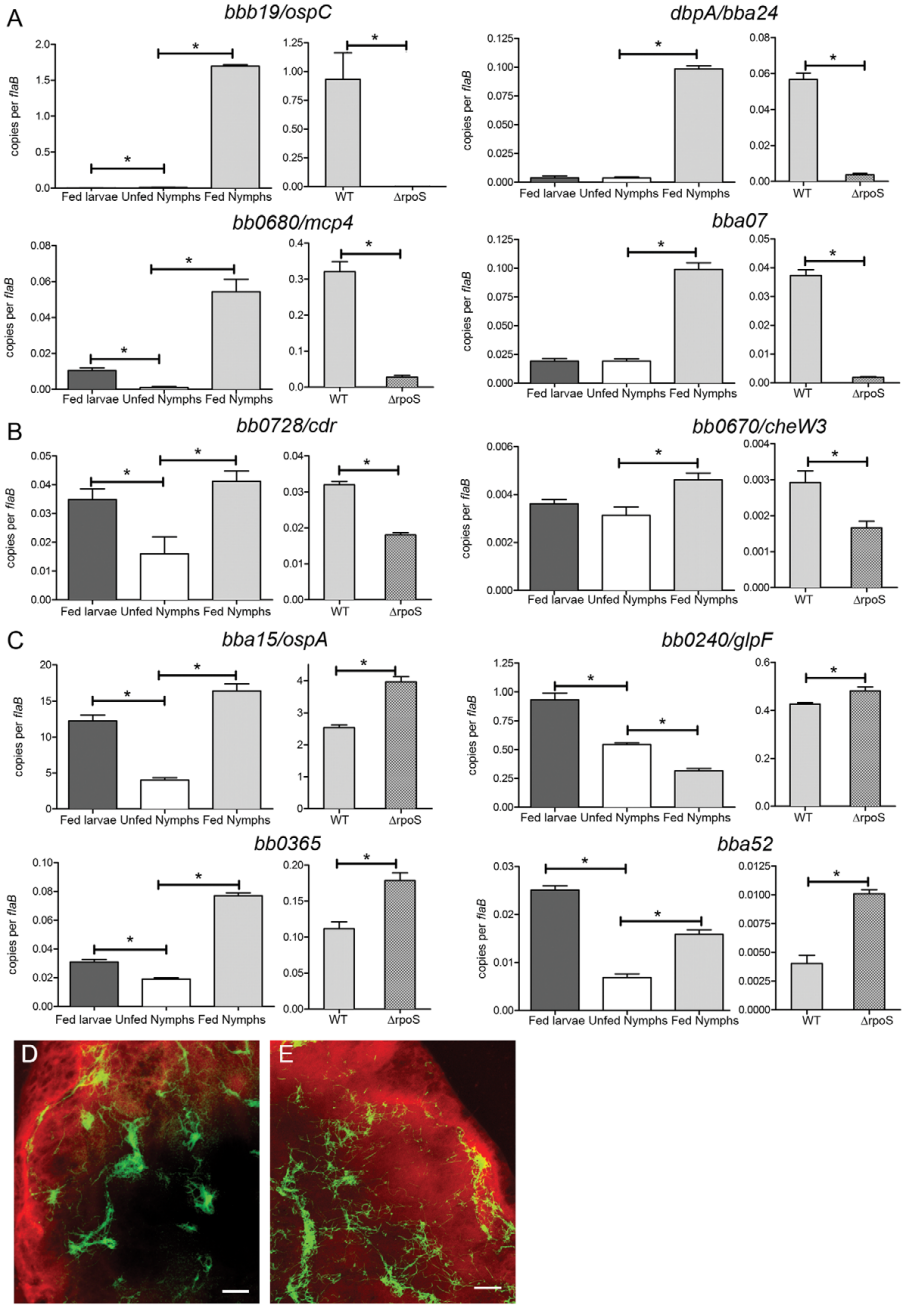
Contours of the RpoS_Bb_ regulon in *I. scapularis*. qRT-PCR analysis of (A) absolutely and (B) partially RpoS-dependent upregulated genes and (C) RpoS-repressed genes selected from microarray data derived from *Bb* cultivated within DMCs [13]. A representative sample of genes is shown; data for the remaining genes are presented in Figure S2. Expression profiling was performed using fed larvae, unfed and fed nymphs that had been naturally-infected with WT *Bb* as well as fed nymphs that had been infected as larvae by immersion with either WT-*gfp* or Δ*rpoS-gfp* isolates. Values represent the average *flaB*-normalized transcript copy number ± standard error of the mean (SEM) for each gene; values are considered significantly different when *p* is ≤0.05 (indicated by asterisks). Composite confocal images through the full thickness of nymphal midguts at 72 h post-placement showing the distribution of spirochetes expressing *gfp* under the control of the (D) *flaB* or (E) *ospA* promoter. A detailed schematic indicating how confocal images of fed midguts were acquired is presented in Figure S3. Here and elsewhere, green represents GFP^+^ spirochetes while red indicates midgut epithelial cells labeled with FM4-64; scale bars = 25 µm.

The above results established the importance of defining the RpoS regulon during the tick-phase of the enzootic cycle. At the present time, however, delineation of the spirochete's transcriptome within ticks is not technically possible. Therefore, we used qRT-PCR to measure transcript levels for a panel of 10 RpoS-upregulated and 6 RpoS-repressed tick-phase genes derived from our microarray analysis of WT and *ΔrpoS* spirochetes cultivated within dialysis membrane chambers (DMCs) [13]. Representative results are shown in Figure 1, while results for the remainder of the panel are presented in Figure S2. All eight genes previously shown to be absolutely RpoS-dependent in DMC-cultivated spirochetes were induced during nymphal feeding with little to no transcript detectable in either fed larvae or unfed nymphs. Two of the upregulated *Borrelia* genes in our panel (*bb0728/cdr* and *bb0670/cheW3*) were selected because they were transcribed by both RpoS and σ^70^ [13]. In accord with these findings, transcripts for both genes were significantly enhanced in fed compared to unfed nymphs. It was interesting to note, however, that in fed larvae, when RpoS is OFF, both *cdr* and *cheW3* were expressed at levels comparable to those in fed nymphs. Particularly noteworthy, all six tick-phase genes repressed by RpoS in the mammal were well expressed during the nymphal blood meal; in fact, three (*ospA*, *bb0365*, and *bba52*) were expressed at their highest levels within fed nymphs.

To corroborate the above expression data, we also examined the same panel of *Bb* genes in fed nymphs that had been infected as larvae by immersion with either WT-*gfp* (Bb914) or *ΔrpoS-gfp* (Bb1058) isolates. Little to no transcript was detected for 8 of the 10 RpoS-upregulated genes in fed nymphs infected with *ΔrpoS-gfp* organisms compared to their WT-*gfp* counterparts (Figures 1A and S2A). Expression of *cdr* and *cheW3* was reduced significantly in nymphs infected with the mutant compared to WT, establishing definitively that these genes are partially-dependent on RpoS for expression during the nymphal blood meal (Figure 1B and [29]). Consistent with results obtained using naturally-infected ticks, the genes repressed by RpoS in mammalian host-adapted organisms were expressed at equal or higher levels by *ΔrpoS-gfp* spirochetes compared to WT-*gfp* (Figures 1C and S2B). Confocal microscopy of WT spirochetes harboring fluorescent transcriptional reporters for *flaB* or *ospA* was performed as an additional means of confirming that RpoS-repressed tick-phase genes are expressed throughout the nymphal blood meal. As shown in Figures 1D and 1E, the overall density and distribution of the two reporter strains within fed nymphal midguts were indistinguishable. Collectively, our findings indicate that genes whose expression is upregulated by RpoS are likely to be expressed both during and after transmission by nymphs while RpoS-dependent repression appears to occur only after spirochetes have fully adapted to the mammalian host.

### Spirochetes introduced into larvae by immersion undergo biphasic dissemination during nymphal feeding

Lyme disease spirochetes disseminate through the midguts of naturally-infected nymphs in two stages: an initial adherence-mediated migration phase during which non-motile spirochetes advance as networks toward the basolateral surface of the epithelium, followed by a transition into motile organisms at or near the basement membrane which then access the hemocoel [30]. The data in Table 2 suggested that spirochetes lacking RpoS are defective in one or both of these phases. In order to examine the contribution of RpoS to biphasic dissemination, we first established that transmission of WT-*gfp Bb* by nymphs infected as larvae by immersion recapitulates the sequence of events previously described for naturally-infected nymphs [30]; a detailed schematic indicating how confocal images of unfed and fed midguts were acquired is presented in Figure S3. Spirochete burdens in unfed and fed nymphs infected by immersion were comparable to those detected in nymphs infected by the natural route (Figure S4A; *p* = 1.000). By confocal microscopy, the distribution of spirochetes within the midguts of unfed and feeding nymphs infected by either method were highly similar (Figure S4B and [30]). The kinetics of transmission by nymphs infected by either route, based on the recovery of organisms in hemolymph, also were indistinguishable (Table 2 and [30]).

### *ΔrpoS* organisms are confined to the midgut lumen and develop into an aberrant morphotype during nymphal feeding

Having confirmed the suitability of the immersion method, we proceeded to characterize Δ*rpoS* spirochetes within nymphal midguts. For these experiments, we generated fluorescent versions of our previously characterized Δ*rpoS* mutant CE174 (Δ*rpoS-gfp*; Bb1058) and complemented strain (Δ*rpoS-gfp*+*rpoS*; SE186) (Table 1 and Figure S5). At the outset, we confirmed using qPCR and semi-solid plating that Δ*rpoS-gfp Bb*, like its non-fluorescent counterpart, replicated to wild-type levels within feeding nymphs (Figure S1B) but was unable to traverse the midgut or establish infection (Table 2). As expected, the complement was fully virulent by tick- and needle-inoculation (Tables 2 and S2).

**Figure 2:**
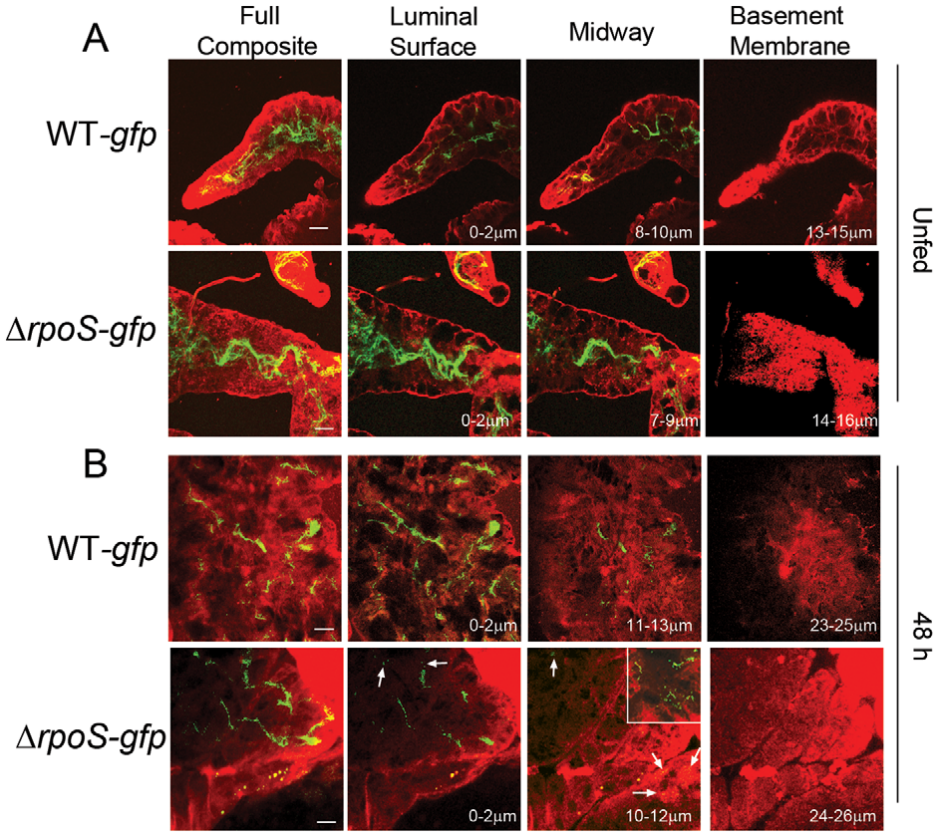
Spirochetes lacking RpoS are distributed normally within unfed nymphal midguts but are destroyed between epithelial cells early during feeding. The leftmost images in each panel depict the full composites (basement membrane to basement membrane) of the midgut, while 3-µm composite images show spirochetes at the luminal surface, midway through the epithelium, and at the basement membrane; scale bars = 25 µm. A detailed schematic indicating how confocal images of unfed and 48 h-fed midguts were acquired is presented in Figure S3. (A) In unfed midguts, WT-*gfp* and *ΔrpoS-gfp* are similarly distributed. (B) In midguts isolated at 48 h post-placement, WT-*gfp* organisms form aggregates, while *ΔrpoS-gfp* located between epithelial cells are destroyed; arrows indicate ragged, blebbing *Bb*. Numbers in lower right hand corner indicate the optical depth of the image. Inset in the *ΔrpoS-gfp* midway panel depicts a 1-µm optical section showing thin, ragged and blebbing organisms between epithelial cells; see also Figure S4 for a comparison of midguts infected with WT-*gfp Bb* by natural versus immersion methods.

**Figure 3:**
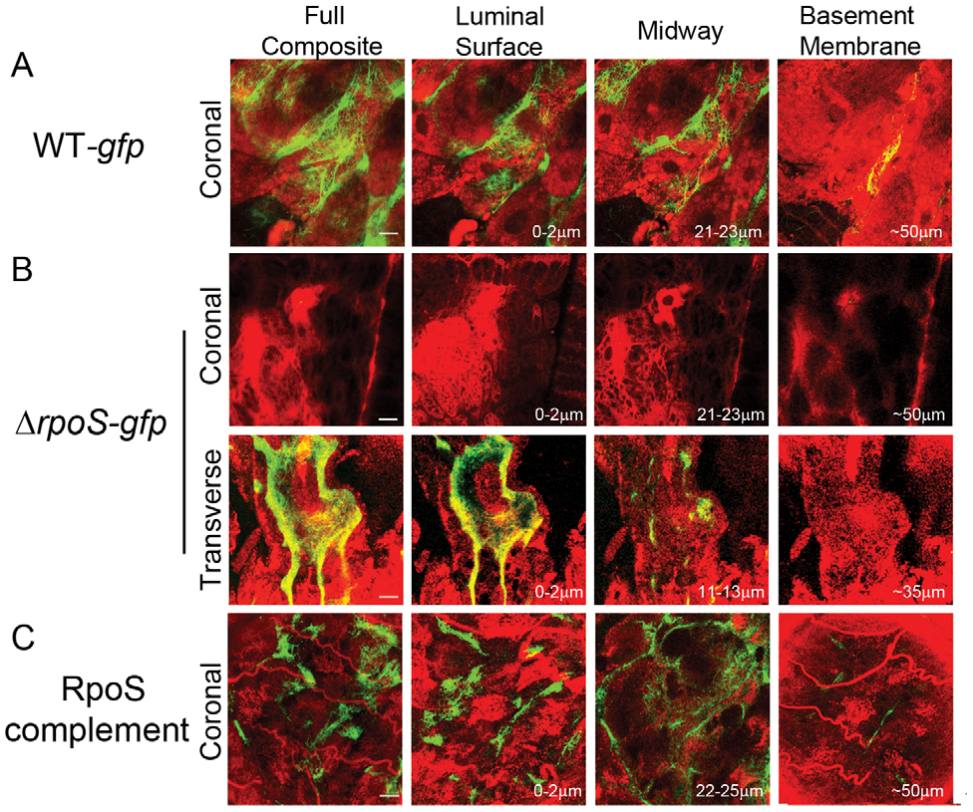
RpoS is required to form networks during nymphal feeding. (A) At 72 h post-placement, WT-*gfp* organisms form networks that encase midgut epithelial cells and extend to the basement membrane. (B) *ΔrpoS-gfp Bb* are not observed in intact midguts imaged in the ‘coronal’ plane but are visualized within the lumens of midguts that had been punctured to remove a portion of their contents and imaged by acquiring transverse optical sections from distal portions of the diverticula to reduce light scattering and absorption by the blood meal. A detailed schematic illustrating how coronal and transverse confocal images were acquired is presented in Figure S3. (C) Complementation restores the ability of *ΔrpoS-gfp Bb* to form networks and extend to the basement membrane; see also Figure S5 for complementation verification by protein profile analysis. The leftmost images in each panel depict the full composites (basement membrane to luminal surface) of the midgut, while 3-µm composite images show spirochetes at the luminal surface, midway through the epithelial layer, and at the basement membrane. Numbers in lower right hand corner indicate the optical depth of the image; scale bars = 25 µm. Representative cryosections of 72 h-fed midguts infected with WT-*gfp* and *ΔrpoS-gfp* isolates are presented in Figure S6.

**Figure 4:**
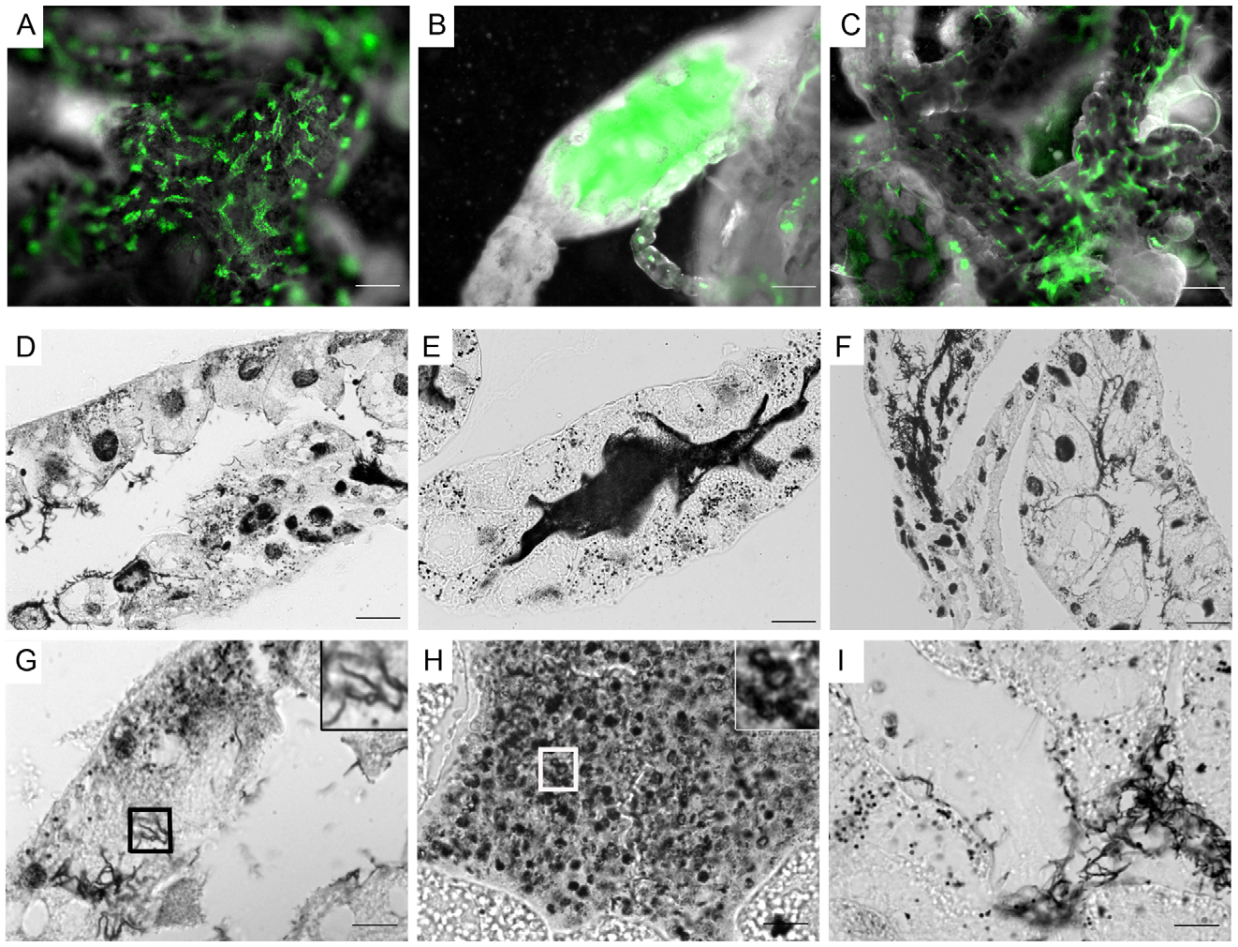
Spirochetes lacking RpoS are confined to the lumen and develop into round bodies during the later stages of feeding. (A–C) Representative epifluorescence images of midguts isolated at 72 h post-placement containing (A) WT-*gfp*, (B) *ΔrpoS-gfp* or (C) RpoS-complemented isolates; midguts appear white when imaged by dark-field microscopy. (D–I) Representative silver-stained paraffin-embedded sections of midguts isolated from 72 h-fed nymphs infected with (D and G) WT-*gfp*, (E and H) *ΔrpoS-gfp* or (F and I) RpoS-complemented isolates. Insets in (G) and (H) are enlargements of the boxed regions showing WT-*gfp* spirochetes and *ΔrpoS-gfp* round bodies, respectively; scale bars: A–C = 50 µm; D–F = 25 µm; and G–I = 10 µm.

By confocal microscopy, the distribution of Δ*rpoS-gfp Bb* in midguts isolated from unfed nymphs did not differ from that of WT-*gfp* organisms (Figure 2A; also see Figure S3). By 48 h post-placement, however, there was a marked reduction in the overall density of Δ*rpoS-gfp Bb* on the luminal surfaces and between epithelial cells (Figure 2B). In addition, many of the mutant organisms located between cells were thin, ragged and blebbing (Figure 2B, ‘midway’ panel). At 72 h, the difference between midguts harboring WT-*gfp* and Δ*rpoS-gfp* isolates was striking (Figure 3). Whereas WT-*gfp* organisms formed typical confluent networks (Figure 3A; WT-*gfp*), Δ*rpoS-gfp Bb* were rarely observed (Figure 3B, Δ*rpoS-gfp* ‘coronal’), a result ostensibly discordant with the comparable spirochete burdens detected in fed nymphs infected with either strain (Figures S1B and S1C). We conjectured that Δ*rpoS-gfp* organisms were confined to the lumen at the late feeding time point but that the blood meal was interfering with their visualization. We confirmed this assumption by carefully removing a portion (∼30–40%) of the blood meal from isolated 72 h-fed midguts and acquiring transverse optical sections from distal portions of diverticula to reduce light scattering and absorption by the luminal contents (Figures S3B–3D). As shown in Figure 3B (Δ*rpoS-gfp*, transverse panel), the lumens of Δ*rpoS*-*gfp*-infected midguts were, in fact, crowded with organisms. Epifluorescence microscopy of intact and cryosectioned midguts (Figures 4B and S6B, respectively) and silver-staining of paraffin-embedded specimens (Figures 4E and 4H) also revealed lumens packed with Δ*rpoS*-*gfp* spirochetes at the 72 h time point. Importantly, complementation restored the ability of Δ*rpoS*-*gfp Bb* to form networks (Figures 3C, 4C, 4F and 4I).

**Figure 5:**
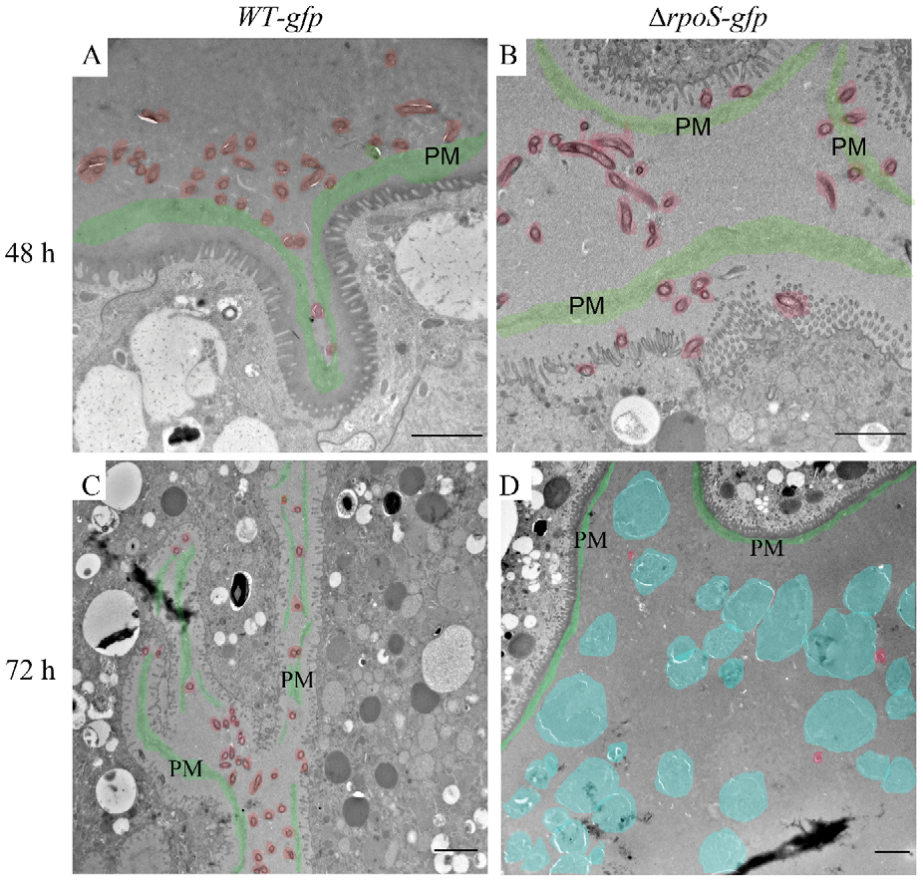
*ΔrpoS Bb* form round bodies within nymphal midguts during the later stages of feeding. Representative TEM images of nymphal midguts containing WT-*gfp* and *ΔrpoS-gfp* spirochetes isolated at (A–B) 48 or (C-D) 72 h post-placement. Color overlays are used to highlight normal spirochetes (red), round bodies (blue), and peritrophic membranes (PM, green); scale bars = 2 µm.

**Figure 6:**
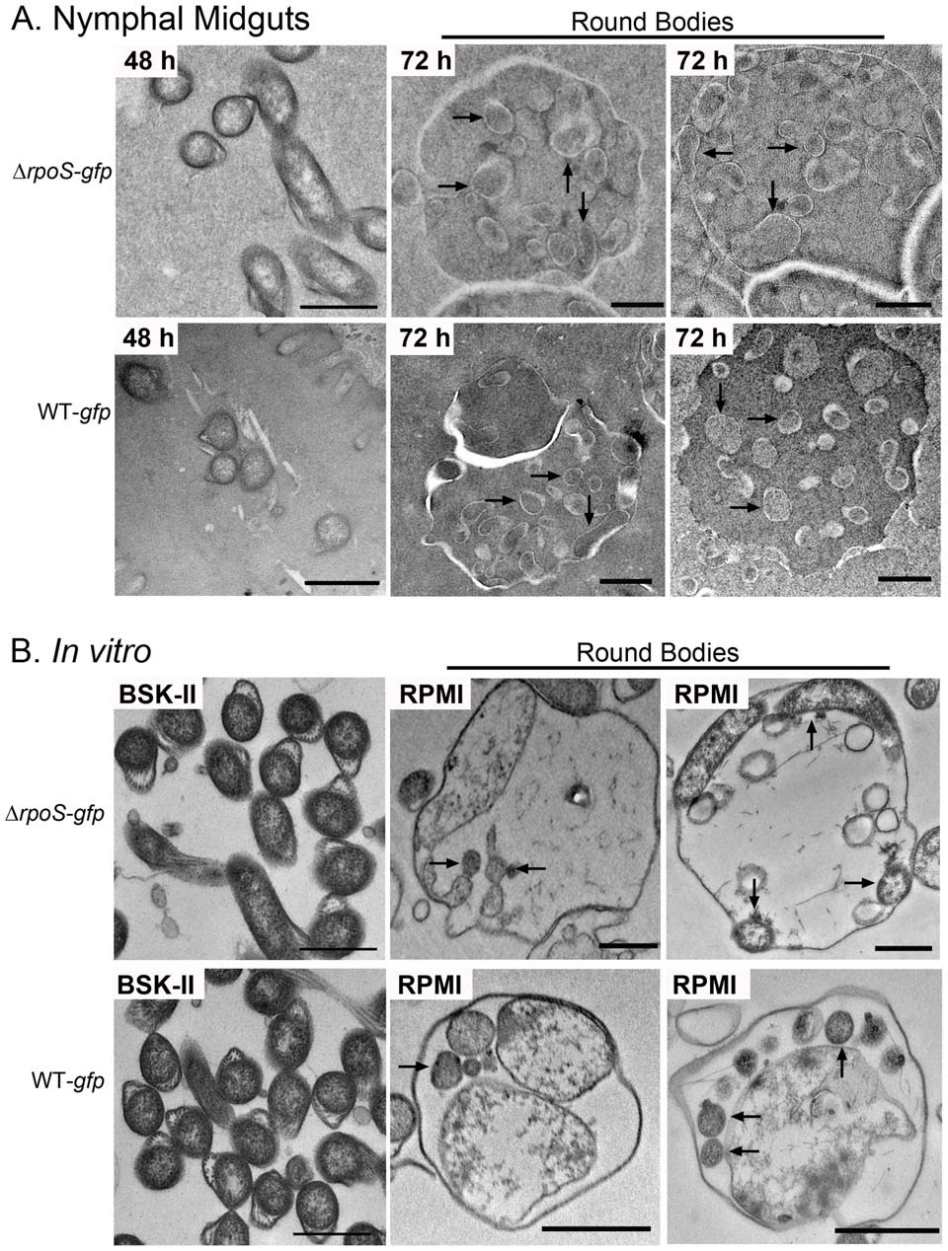
Wild-type and *ΔrpoS* round bodies are structurally similar both within feeding nymphs and under nutrient-limiting conditions *in vitro*. Representative TEM images of *ΔrpoS-gfp* and WT-*gfp* spirochetes and round bodies in (A) nymphal midguts isolated at 48 or 72 h post-placement or (B) *in vitro*-derived organisms following incubation in BSK-II or RPMI for 3 days. In panels A and B, arrows indicate examples of protoplasmic cylinders; scale bars = 500 nm.

Closer inspection of silver-stained paraffin sections at high magnification revealed that the vast majority of Δ*rpoS-gfp* organisms no longer displayed typical spirochete morphology but, instead, were round and larger in diameter than a typical spirochete in cross-section (compare insets in Figures 4G and 4H). We next used transmission electron microscopy (TEM) of ultra-thin sections to better characterize this aberrant morphotype. At 48 h post-placement, there was no discernible difference between the populations of WT-*gfp* and Δ*rpoS*-*gfp* organisms (Figures 5A and 5B). By 72 h post-placement, however, a striking difference was readily apparent (Figures 5C and 5D). In contrast to WT organisms, Δ*rpoS*-*gfp Bb* were predominantly membranous, spherical structures containing irregularly-shaped vesicles and protoplasmic cylinders (Figures 5C, 5D, and 6A). Based on the nomenclature proposed by Brorson *et al.* [23], we hereafter use the term ‘round bodies’ to denote these structurally heterogeneous entities. Interestingly, WT round bodies also were observed sporadically in midguts at the 72 h time point, examples of which are shown in Figure 6A.

### Loss of RpoS and CoADR exacerbate the formation of round bodies *in vitro*

**Figure 7:**
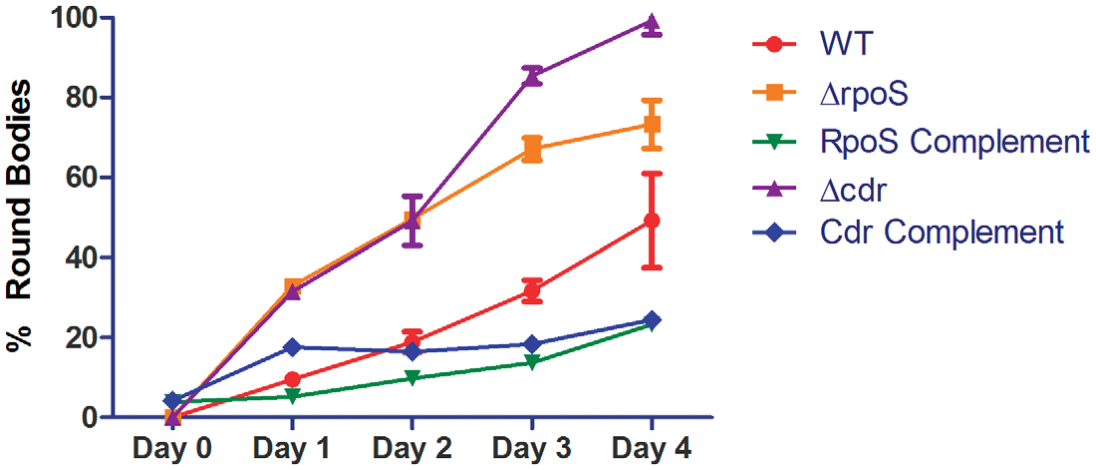
Loss of RpoS and CoADR exacerbates round body formation *in vitro*. *Bb* were incubated in RPMI for 1–4 days. A minimum of 300 organisms were counted per strain for each time point. Experiments were performed in triplicate; error bars represent means ± SEM. Representative images of fields used to quantify round body formation are shown in Figure S7. The percentages of round bodies formed by Δ*rpoS-gfp* and Δ*cdr* isolates were significantly greater than WT on days 1 through 4 (*p*≤0.002). Round body formation by Δ*rpoS-gfp* and Δ*cdr* isolates was significantly different on days 3 and 4 (*p*≤0.002).

Spirochetes cultivated under nutrient-limiting conditions form morphotypes [22]–[24], [31], [32] resembling the round bodies observed in nymphal midguts. These reports led us to hypothesize that RpoS-deficiency creates a metabolic lesion that promotes the formation of round bodies during the nymphal blood meal. To test this idea, we took advantage of the observation by Alban *et al.* [32] that spirochetes form round bodies during a relatively short incubation period in RPMI. As shown in Figure 7, the percentage of WT-*gfp* round bodies increased steadily over 4 days, reaching a plateau of ∼50% on day 3; a significantly higher percentage of Δ*rpoS-gfp* spirochetes formed round bodies on days 1 through 4 (Figure 7, *p*≤0.002 for each day). Complementation of Δ*rpoS-gfp* significantly reduced the percentages of round bodies to levels lower than those observed with WT-*gfp* on days 3 and 4 (Figures 7 and S7, *p*≤0.01 for both days). Moreover, we confirmed by TEM that round bodies formed by WT-*gfp* and Δ*rpoS-gfp* isolates in RPMI were identical to each other (Figure 6B) and closely resembled those in nymphal midguts (Figure 6A). Previously, Xu *et al.* [33] proposed that Δ*ospC* spirochetes have a defective membrane surface. It was thus surprising to find that the percentage of Δ*ospC* round bodies was actually lower than WT levels at days 3 and 4 and that the rate of round body formation was comparable to that observed for WT-*gfp* organisms (data not shown).

In *Bb*, the enzyme CoADR, encoded by *cdr*, contributes to the regeneration of NAD^+^ for glycolysis and the maintenance of the pool of Coenzyme A for biosynthetic functions [34], [35]. Our finding that transcription of *cdr*, one of the few metabolic genes within the RpoS regulon, is markedly reduced in Δ*rpoS-gfp Bb* during nymphal feeding (Figure 1B) suggested that diminished levels of CoADR might be involved in the enhanced formation of round bodies by Δ*rpoS-gfp* in RPMI. As shown in Figure 7, the percentages of round bodies formed by Δ*cdr* and *ΔrpoS-gfp Bb* were not significantly different on days 1 and 2 (*p*>0.6 for both days); by days 3 and 4, however, the rate of round body formation by *Δcdr* organisms significantly outpaced both WT and *ΔrpoS* spirochetes (*p*≤0.0006 and *p*<0.002 for WT and *ΔrpoS*, respectively, on both days). As with Δ*rpoS-gfp Bb*, complemented Δ*cdr* spirochetes formed round bodies at levels lower than those of WT-*gfp* on days 3 and 4 (Figures 7 and S7, *p*≤0.002 on both days).

### Round bodies elongate rapidly upon addition of BSK-II

Alban *et al.* [32] noted that round bodies formed in RPMI elongate within seconds following the addition of normal rabbit serum (NRS). We sought to confirm this observation, which suggests that neither the metabolic lesion that triggers round body formation nor the resulting rearrangement of the spirochete's protoplasmic cylinder involve alterations in the cell envelope that require extensive biosynthetic repair. In our hands, the addition of NRS did not promote round body recovery. However, the addition of an equal volume of Barbour-Stoenner-Kelly medium (BSK-II), either with or without NRS, induced the rapid (≤3 s) recovery of WT-*gfp* and *ΔrpoS-gfp* round bodies (Video S1). Interestingly, one or two highly refractile inclusions often could be observed within round bodies formed in RPMI by both *Bb* isolates; these structures remained prominent even after bacteria had fully elongated. Based on visualization of 32 round bodies formed by WT-*gfp* and *ΔrpoS-gfp Bb* (16 per isolate) in RPMI, we found that each recovered into a single spirochete (Video S1).

**Figure 8:**
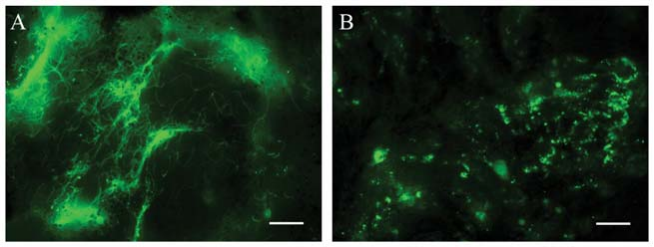
Round bodies within fed nymphal midguts recover into elongated spirochetes. *ΔrpoS*-infected nymphs were removed at 72 h post-placement and midguts dissected into RPMI. (A) The addition of BSK-II induced the recovery of round bodies into elongated spirochetes. (B) Round bodies do not recover when midguts were submerged in RPMI. Scale bars = 10 µm. See also the Video S1 of the rapid recovery of *in vitro*-derived organisms.

To further the comparison between round bodies formed within ticks and RPMI, we next investigated whether round bodies within midguts isolated at 72 h post-placement also recover upon the addition of BSK-II. Indeed, as shown in Figure 8A, tick-derived *ΔrpoS* round bodies elongated into spirochetes within minutes. Importantly, the addition of RPMI did not promote recovery (Figure 8B).

## Discussion

Studies involving RpoS_Bb_ have focused primarily on the contribution of RpoS-dependent genes to virulence in mammals [21]. The role of RpoS_Bb_ during the tick-phase of the enzootic cycle, on the other hand, has been largely unexplored despite multiple lines of evidence suggesting that genes controlled by this alternative sigma factor might function during tick-to-mammal transmission [12], [13], [36]. In this study, we demonstrate that Δ*rpoS* spirochetes remain confined to the midgut throughout the blood meal, and, thus, are unable to progress beyond the initial stage of dissemination. Unexpectedly, RpoS deficient spirochetes replicating within the midguts of feeding nymphs undergo a dramatic morphologic transformation comparable to that induced by nutritional deprivation *in vitro*. The high degree of phylogenetic divergence between RpoS_Bb_ and its orthologs in Proteobacteria [37], the ostensible lack of overlap between the RpoS regulons in *Bb* and *E. coli* [13], and the finding that RpoS_Bb_ does not protect spirochetes against “conventional” external stressors (e.g., reactive oxygen species, low pH, high salt) [27] previously led us to conclude that RpoS_Bb_ is not a central regulator of a general stress response [13]. The findings presented here, however, argue that RpoS-mediated alterations in the spirochete's transcriptome include physiologic adaptations required for dissemination within the vector.

*ospC* is widely regarded as the prototypical RpoS-dependent gene [12]. While there is universal agreement that OspC is essential for the ability of *Bb* to establish murine infection, there is lingering controversy as to whether this surface lipoprotein functions within the vector or the mammal [38]. Pal *et al.* [25] reported that spirochetes lacking OspC were unable to penetrate the salivary glands of feeding nymphs. Rosa and colleagues [19], [26], on the other hand, visualized *ΔospC* organisms within salivary glands and demonstrated that OspC was required for survival within mice following needle-inoculation. Our data, derived entirely by tick-inoculation, are in accord with those of the Rosa group. We found that spirochetes lacking OspC were recovered from hemolymph and reached the tissue surrounding the bite site with kinetics indistinguishable from those of WT *Bb* but were cleared from the skin within 48–72 h post-repletion, a time frame which closely mirrors that previously reported using needle-inoculation [26]. The inability of Δ*rpoS Bb* to traverse the midgut, therefore, cannot be attributed to the lack of OspC. We recently reported that spirochetes lacking *cdr* possess a growth defect within feeding nymphs and are avirulent by needle inoculation [29]. Thus, RpoS-dependent genes fall into three broad categories: (i) *ospC*-like genes that are required solely within the mammal; (ii) genes, such as *bba64* [39], [40], that promote dissemination within the tick; and (iii) genes, such as *cdr*, that function in the arthropod as well as the mammal [29].

The qRT-PCR studies described herein brought to light a striking difference in the transcriptional profiles of WT *Bb* within larval and nymphal ticks as compared to mammalian host-adapted (i.e., DMC-cultivated) organisms: while genes upregulated by RpoS in DMCs also were induced in fed nymphs, genes repressed by RpoS in mammals were expressed at comparable levels by *Bb* in all tick life stages. A comparison of transcript copy numbers in WT and Δ*rpoS Bb* demonstrated that several tick-phase genes (i.e., *ospA*, *bb0240*, *bb0365*, and *bba52*) are, in fact, subject to some degree of downregulation during the nymphal blood meal. One possible mechanistic explanation for the limited RpoS-mediated repression observed within fed nymphs is that signals produced during the blood meal activate regulatory pathways that oppose the downregulation of tick-phase genes during transmission. The Hk1/Rrp1 two-component system, which promotes tick-adaptation during feeding but is not required for mammalian infection [41]–[43], is a candidate counter-regulatory pathway. Our findings add to a large body of evidence that the “classical” paradigm for reciprocal regulation of tick- and mammalian host-phase genes (*ospA*/*ospC* being the prototypes) is valid only within the mammal [13], [14], [16], [44], [45]. A number of recent studies have helped to elucidate the biological significance for the prolonged transition from the tick- to the mammalian host-adapted state [46]–[51]. As one example, *Bb* requires the products of the *glp* operon (*bb0240-0243*) to maintain maximal fitness throughout all tick life stages [51] because these gene products enable spirochetes to import and utilize glycerol, an increasingly important carbon source within the arthropod as the availability of glucose diminishes during digestion of the blood meal. In addition, the continued expression of tick-phase borrelial surface adhesins, such as OspA [17], [52], along with the inhibition of motility by components elaborated by the midgut during the nymphal blood meal, may facilitate the close association of *Bb* with midgut epithelium, a prerequisite for network formation and adherence-mediated migration [30]. Maintaining a large population of organisms that are both tick- and mammalian host-adapted could be advantageous for *Bb* by enhancing the probability that the small number of spirochetes that reach the basement membrane are “transmission ready” while ensuring that the large number of organisms remaining in the midgut can resume the fully tick-adapted state post-repletion.

As a prelude to defining the contribution of RpoS during the tick-phase of the enzootic cycle, we first confirmed that the transmission of spirochetes by ticks infected by immersion faithfully reproduces the spatio-temporal sequence of events previously described for organisms acquired naturally [30], [53]. Discernible differences between WT-*gfp* and *ΔrpoS-gfp Bb*, first apparent at 48 h post-attachment, were dependent upon the positioning of *ΔrpoS-gfp* spirochetes relative to the epithelial cells. The degradation of *ΔrpoS-gfp Bb* between cells suggests that one or more RpoS-dependent genes are required for protection against noxious elements encountered in the intercellular spaces. That spirochete burdens in fed nymphs infected with *ΔrpoS Bb* were not significantly diminished suggests that the destruction of organisms is confined to a small niche within the midgut. Another contrast between WT and mutant *Bb* at the 48 h time point was the paucity of *ΔrpoS-gfp* organisms at the luminal surface. Given that RpoS-deficient *Bb* accumulate within the lumen as feeding progresses, one might attribute this difference to an attachment defect associated with loss of RpoS-upregulated surface molecules that function cooperatively with tick-phase adhesins. Bioinformatic analysis of the RpoS regulon revealed 38 potential surface adhesins (Table S3); seven are predicted outer membrane-spanning proteins (i.e., β-barrels) based on the computational consensus framework used by Cox *et al.* [54] while the remainder are lipoproteins [55].

The most striking aspect of the Δ*rpoS* phenotype, the transformation from typical spirochetes to round bodies, did not become apparent until 48–72 h post-attachment. During this late stage of feeding, the nutritional requirements of midgut epithelial cells immensely increases as the cells switch from undifferentiated reserve cells into hyperactive digestive cells, while spirochetes replicate extensively around them [30], [56]. Presumably, an intense competition for blood meal nutrients ensues at this interface. Within this same 24 h window, a small percentage of organisms that reach the basement membrane become motile, a transition that enables them to access the hemocoel but likely also substantially adds to their energy requirements. Spirochetes lacking RpoS replicate to WT levels within the midguts of feeding nymphs and exhibit growth kinetics equivalent to those of WT as demonstrated by qPCR and semi-solid plating. These findings argue strongly that the formation of round bodies by Δ*rpoS Bb* cannot be attributed to their inability to utilize the blood meal for biosynthetic purposes but rather suggest that loss of RpoS creates a rapidly reversible metabolic lesion that provokes the structural rearrangements that give rise to round bodies. The ability of *ΔrpoS* round bodies derived from tick midguts to recover within minutes after the addition of BSK-II (i.e., without replication) strongly supports this contention. To shed light on this unusual morphotype, we turned to an *in vitro* system in which WT *Bb* transform into round bodies during several days of incubation in RPMI [32]. Although the culture conditions obviously differ considerably from the nutrient-replete midgut, the round bodies formed by WT and *ΔrpoS Bb in vitro* closely resemble those observed in feeding nymphs. Several other features of this *in vitro* system are particularly instructive and support its utility for elucidating the *in vivo* morphotype. First, round bodies elongate rapidly in the presence of BSK-II, indicating that their cell envelopes are intact. Second, spirochete numbers do not increase in this system, demonstrating that round body formation is divorced from replication. Third, and most importantly, compared to WT organisms, Δ*rpoS Bb* exhibited a pronounced tendency to form round bodies, suggesting that the metabolic lesion in feeding nymphs is operative *in vitro*. This supposition led us to examine *cdr*, whose enzymatic product, CoADR, provides the cofactor NAD^+^ for glycolysis as a byproduct. While the *Δcdr* phenotype provides proof of principle that an energy-related gene product is involved in round body formation, additional genes within the RpoS regulon also may contribute to this phenomenon.

A fundamental question posed by this work is whether round body formation is a unique stress response to nutrient limitation or an aberration of the laboratory with no role in the natural ecology of the spirochete. Formation of round bodies by WT spirochetes argues for its biological relevance as does the sheer complexity of the process, its elegant recovery mechanism, and the stress-related circumstances in which it is observed. Round body formation by WT spirochetes in RPMI can be conceptualized as a response to inadequate energy generation. In contrast, round body formation by WT spirochetes within the midguts of feeding nymphs is sporadic; perhaps the competition between spirochete and epithelial cells for micro-nutrients in the blood meal is particularly intense in localized regions of the midgut. What is clear is that, in both settings, physiologic adaptations mediated by RpoS inhibit round body formation. This formulation implies that (i) the apparatus for round body formation and recovery is poised to operate within the background during feeding but is suppressed either directly or indirectly by an RpoS-dependent gene product and (ii) spirochetes default to round bodies upon sensing that the energetics for transmission (i.e., transition to motility) are unfavorable. Underlying many studies of round body formation in the literature has been the presumption that its primary importance relates to persistence within the mammal [22], [24], [31]. We propose that round body formation has evolved to support the tick phase of the cycle and predict that there are circumstances, as yet undefined, when spirochetes within the tick resort to this survival program on a large scale in order to maintain a population of transmissible organisms.

## Materials and Methods

### Ethics statement

All animal experimentation was conducted following NIH guidelines for housing and care of laboratory animals and in accordance with University of Connecticut Health Center regulations after review and approval by the UCHC Institutional Animal Care and Use Committee.

### Culture and maintenance of bacterial strains

*B. burgdorferi* isolates used in these studies (Table 1) were cultivated in modified BSK-II [57] supplemented with 6% rabbit serum (Pel-Freeze Biologicals). Selection and maintenance of *B. burgdorferi* strains was performed in media supplemented with the appropriate antibiotics (Table 1). The plasmid content of all isolates was monitored as previously described [58]. Standard temperature-shift experiments and growth curves were performed as previously described [13]. *Escherichia coli* strains were maintained in Luria-Bertani broth (LB) (1% tryptone, 0.5% yeast extract, 1% NaCl) with the appropriate antibiotic. Selection was performed on LB agar plates (LB with 1.5% agar) supplemented with the appropriate antibiotic.

### DNA manipulations and routine cloning

Routine molecular cloning and plasmid propagation were performed using *E. coli* Top10 cells (Invitrogen). Routine and high-fidelity PCR amplification reactions were performed using Choice *Taq* (Denville Scientific) and Takara ExTaq (Fisher Scientific), respectively. Plasmid DNAs were purified using Qiagen Midi and Spin Prep kits. Nucleotide sequencing was performed by Agencourt Bioscience Corp.

### Strain construction

Strains CE162, CE174, CE467, CE56, CE103, Bb914, CE309, and CE1655 have been previously described [27], [29], [30], [36], [59] (Table 1). To generate an *ospC* deletion mutant, we amplified overlapping upstream and downstream regions containing *ospC* (*bbb19*) and flanking sequences from strain 297 using primers BBB18#1F/BBB19#1R(NgoMIV) and BBB19#1F(NgoMIV)/BBB22#1R, respectively (Table S1). The resulting amplicons were cloned into pCR2.1-TOPO (Invitrogen), re-amplified using the same primers as above, and gel-purified. Purified upstream and downstream amplicons were mixed 1∶1 and amplified for 4 cycles with ExTaq in the absence of primer to promote annealing/extension of the overlapping regions followed by 30 cycles in the presence of BBB18#1F and BBB22#1R flanking primers. The joined amplicon was subsequently cloned into pCR2.1-TOPO, digested with EcoRI, and subcloned into similarly digested pUC19 to yield pCE216. A P*_flgB_-kan* cassette conferring resistance to kanamycin was amplified from pTAKanG [60] using PflgB(NgoMIV) and KanR(BamHI) and cloned into pCR2.1-TOPO. The P*_flgB_-kan* cassette was liberated by digestion with NgoMIV and BamHI and ligated into NgoMIV-BglII digested pCE216 to yield pCE217. Approximately 10 µg of gel-purified *ospC::kanR* insert, released from pCE217 by digestion with NgoMIV and BamHI, was electro-transformed into CE162 (Table 1) as previously described [61]. A transformant (CE303) was selected in BSK-II medium containing the appropriate antibiotic and subsequently confirmed by PCR to be an *ospC* deletion mutant using the BBB18#1F (extd) and BBB22#1R primers (Table S1). Bb1058, the fluorescent *rpoS* mutant, was generated by electro-transforming CE174 with pMC1916 [30] followed by selection in BSK-II containing erythromycin (0.06 µg/ml) and gentamycin (5 µg/ml). Inactivation of *rpoS* was confirmed by PCR using the primers PrpoS-5′ and rpoS-3 (Table S1). To generate the fluorescent *ΔrpoS* complement, competent Bb1058 cells were transformed with a wild-type copy of *rpoS* contained on pCE320 as previously described [27]. A transformant, SE186, was selected in BSK-II medium containing kanamycin, erythromycin, and gentamycin and subsequently confirmed to contain *rpoS* by PCR using the primers rpoS-prom(extd) and RpoS-3′XbaI (Table S1). Complementation was confirmed by the production of OspC and DbpA following temperature-shift as assessed by silver-stain and immunoblot, respectively, as previously described [27].

### Assessment of *Bb* infectivity by needle inoculation

Infectivity was assessed using 4–6 week old female C3H/HeJ mice (five per group, per isolate) that were inoculated intradermally with 1×10^4^ spirochetes. Infection was assessed at 2 and 4 weeks post-inoculation by serology and cultivation of tissues (ears, skin, joints, hearts, and/or bladders) in BSK-II containing sulfamethoxazole (0.05 mg/ml), phosphomycin (0.02 mg/ml), rifampin (0.05 mg/ml), trimethoprim (0.01 mg/ml) and amphotericin (0.0025 mg/ml). Cultures were monitored weekly by dark-field microscopy.

### Generation of *Bb* infected *I. scapularis* ticks

To generate naturally-infected ticks, 300 to 400 pathogen-free *I. scapularis* larvae (Oklahoma State University) were placed on infected C3H/HeJ mice 2 to 3 weeks after needle inoculation with CE162 as described above; larvae were allowed to feed to repletion and then held in an environmental incubator until they had molted. For experiments using *Bb* isolates that were avirulent by syringe, see above, naïve larvae were infected according to the immersion method described by Policastro and Schwan [28], fed on naïve C3H/HeJ mice, and allowed to molt into nymphs.

### Assessment of *B. burgdorferi* survival by quantitative culture and qPCR

Spirochete burdens were assessed by quantitative PCR (qPCR) using individual pools of 10 unfed and 5 replete nymphs. Total genomic DNA was isolated from surface-sterilized nymphs using the Gentra Puregene Yeast and Bacteria kit (Qiagen) according to the manufacturer's instructions. DNAs were diluted 1∶10 in water and analyzed using a TaqMan-based assay for *flaB* ([30] and Table S1). In some experiments, the viability of spirochetes within replete nymphs (5 per strain) was assessed by semi-solid plating in pBSK medium as previously described [61].

### Assessment of spirochete dissemination in ticks and transmission to mice

Groups of 10 to 12 infected unfed nymphs were confined to capsules affixed to the backs of naïve C3H/HeJ mice [14]. At 72 h post-placement, approximately 5–6 feeding nymphs were forcible removed, surface-sterilized, and hemolymph collected for culturing in BSK-II as previously described [30], while the remaining ticks were allowed to feed to repletion. At 24, 48 and 72 h post-repletion, groups of mice (3–4 per strain, per time point) used as a blood meal source for infected nymphs were sacrificed and their capsules removed. For each mouse, a 3 cm×2 cm region of skin encompassing the feeding site was demarcated using a grid. The excised skin then was cut into equal portions, rinsed in 70% ethanol, and cultured in BSK-II. *Bb* burdens in replete nymphs were determined using qPCR as described above.

### qRT-PCR

For expression studies involving naturally-infected ticks, total RNA was isolated from ∼150 fed larvae or unfed nymphs and ∼75 engorged nymphs that had been infected as larvae by feeding on a mouse infected with CE162. For studies comparing gene expression in WT-*gfp* (Bb914) and Δ*rpoS-gfp* (Bb1058) isolates, total RNA was isolated from ∼60 engorged nymphs that had been infected as larvae by immersion and then fed as nymphs on naïve mice as described above. RNA was isolated from infected ticks using TRIzol reagent (Invitrogen) as previously described [14]. cDNAs (+RT) were assayed in quadruplicate using iQ SYBR Green Supermix (BioRad) and gene specific primers (Table S1). Transcript copy numbers were calculated using the iCycler post-run analysis software based on internal standard curves for each gene. Expression levels for each gene of interest were normalized against copies of *flaB* determined using a TaqMan-based assay (Table S1) performed using iQ Supermix.

### Processing of nymphal midguts for light microscopy

For fluorescence microscopy, intact midguts were isolated from unfed and partially-fed (48 and 72 h post-placement) nymphs and labeled with FM4-64 (2 ng/ml in PBS; Invitrogen) as described previously [30]. Confocal microscopy was performed on a Zeiss LSM-510; serial Z-series images were acquired in both red and green channels [30]. Briefly, our standard methodology for acquiring serial Z-series images involves obtaining 1-µm optical sections through the depth of the midgut that can be resolved by a confocal microscope (∼50 µm). The basement membrane represents the first image in each Z-series; subsequent optical sections were acquired through the epithelial layer towards the lumen (Figure S3A). For midguts isolated from unfed and 48 h-fed nymphs, optical sections were acquired through the full thickness of each specimen (i.e., from basement membrane to basement membrane, Figure S3A). As feeding progresses, influx of the blood meal and expansion of the luminal space precludes our ability to image through the full thickness (>100 µm) of individual diverticula. Consequently, optical sections of 72 h-fed midguts were acquired through a single epithelial layer (i.e., from the basement membrane to the luminal surface, Figure S3B). Representative composites of three consecutive 1-µm optical sections acquired (i) at the luminal surface, (ii) midway through the epithelium, and (iii) at the basement membrane are used to present the distribution of spirochetes through the midgut; the midway point for each specimen was determined during image processing based on the thickness of the epithelial layer. Routine optical sections of unfed and fed midguts were acquired in the coronal plane (Figure S3C). To visualize *ΔrpoS*-*gfp* organisms within 72 h-fed midguts, however, two additional steps were required to reduce light scattering and absorption by the luminal contents. First, we carefully removed a portion (∼30–40%) of the blood meal to minimize interference from the blood meal and reduce the overall thickness of the midgut. Individual diverticula were then imaged in the transverse plane beginning at or near the tip (Figure S3D). A minimum of 20 *Bb*-infected nymphal midguts were examined at each time point. Epifluorescence microscopy was performed on an Olympus BX41 microscope equipped with a Retiga Exi (QImaging) camera; images were acquired using a 40× (1.4 NA) oil immersion objective with QCapture software v. 2.1 (QImaging). Fed midguts also were processed for paraffin-embedding and silver staining using the Wharthin-Starry method as previously described [30].

### Transmission Electron Microscopy (TEM)

For TEM, fed midguts were isolated at 48 and 72 h post-placement and immersed for 3 h at 4°C in 0.1 M sodium cacodylate buffer (CAC) containing 4% paraformaldehyde and 2.5% glutaraldehyde. The midguts then were embedded in 1% agarose solution in 0.1 M CAC buffer. After solidification of the agarose, the midguts were sliced into >1 mm fragments, transferred to fresh 0.1 M CAC buffer and incubated overnight at 4°C. The specimens then were rinsed with fresh 0.1 M CAC buffer and post-fixed for 2 h in 0.1 M CAC buffer containing 1% OsO_4_, and 0.8% potassium ferricyanide. Individual midgut slices were washed in water, stained with 1% uranyl acetate, dehydrated in ascending ethanol solutions, and embedded in PolyBed812. Thin sections (70 nm) were cut on an ultramicrotome (Leica), collected on formvar coated slot grids, and stained in 2.5% uranyl acetate and lead citrate. The stained thin sections were viewed on a transmission electron microscope (Hitachi H-7650) at 80 kV. Images were acquired using an AMT camera and Image Capture Engine v6.01 software (Advanced Microscopy Techniques). All images were processed using ImageJ v.1.42 software [62]. In Figure 5, color overlays were added using Adobe Photoshop (CS4).

### Round body formation and recovery

Serum starvation experiments were performed as previously described [32]. Briefly, aliquots (∼400 µl) of temperature-shifted cultures at late logarithmic phase were transferred to 15 ml of RMPI-1640 (Invitrogen, catalogue #11875) at a final density of 1.5×10^7^ spirochetes/ml) and incubated at 37°C. At 24 h intervals, organisms were enumerated by darkfield microscopy and spirochete morphology was assessed by either darkfield (non-fluorescent isolates) or epifluorescence (fluorescent isolates) microscopy. To assess the ultrastructure of round bodies, spirochetes cultivated in RPMI or BSK-II for 3 days were centrifuged at 6800 g, rinsed in PBS, and resuspended in 0.1 M CAC buffer containing 4% paraformaldehyde and 2.5% glutaraldehyde. The organisms were incubated on ice for 15 minutes then centrifuged at 8000× g. Cell pellets were embedded in agarose and processed for examination by TEM as described above. *In vitro* generated round bodies were recovered by the addition of an equal volume of BSK-II after 4 days of cultivation in RPMI. Recovery was monitored in real-time using StreamPix software (Norpix) on an Olympus BX41 microscope equipped with a Retiga Exi camera. Videos were generated using QuickTime Player v.7.7 (Apple, Inc.). The recovery of round bodies in nymphs at 72 h post-placement was performed by first dissecting the midgut in RPMI. The diverticula were then detached from the stomach using a scalpel blade. A cover slip was then applied to the cover glass and real-time imaging was performed for the RPMI control. Round body recovery in nymphal midguts was induced by adding 10 µl BSK-II to the dissected midguts. Recovery was monitored in real-time as described above.

### Bioinformatics

Candidate surface-exposed lipoproteins within the RpoS regulon [13] were identified using the database created by Setubal *et al.* [55]. Non-surface exposed lipoproteins then were excluded based on published data [49], [63], [64]. Candidate RpoS-dependent β-barrel-outer membrane spanning proteins (OMPs) were identified and filtered using the consensus computational framework recently used to identify OMPs in *Treponema pallidum* [54]. The proteins identified by two or more OMP localization/topology programs were designated OMP candidates (Table S3).

### Statistical analysis

Statistical analyses were performed on qPCR, spirochete viability, and round body formation using Prism 5 (GraphPad Software, Inc.). Student's *t* tests were used to calculate the significance of the qPCR and spirochete viability (*p*≤0.05 was considered significant) and is indicated in the figures and/or legends. Unpaired student's t test with two-tailed *p* values were used to compare round body formation between different *Bb* isolates at each of the time points studied. For qRT-PCR, the normalized copy number values for each gene of interest were compared in Prism using an unpaired *t*-test with two-tailed *p* values and a 95% confidence interval.

## Supporting Information

Figure S1 **Spirochete persistence and survival in** ***I. scapularis*** **are not affected by loss of either RpoS or OspC.** (A) Spirochete burdens of WT, *ΔrpoS*, RpoS complemented RpoS Compl) and *ΔospC* before and after feeding on naïve C3H/HeJ mice. The nymphs used in these experiments were used in Table 2. WT-*gfp* and *ΔrpoS-gfp* (B) burdens determined by qPCR and (C) viability, assessed by semi-solid plating, are highly similar in unfed (black) and fed (gray) nymphs. Values represent the means ± SEMs from three independent experiments.
(TIF)

Figure S2 **Contours of the RpoS_Bb_ regulon in** ***I. scapularis*** **.** qRT-PCR analysis of (A) absolutely and (B) partially RpoS-dependent upregulated genes selected from microarray data derived from *Bb* cultivated within DMCs [13]. Expression profiling was performed using fed larvae, unfed and fed nymphs that had been naturally-infected with WT *Bb* as well as fed nymphs that had been infected as larvae by immersion with either WT-*gfp* or Δ*rpoS-gfp* isolates. Values represent the average *flaB*-normalized transcript copy number ± SEM for each gene; average values are considered significantly different when *p* is ≤0.05 (indicated by asterisks).
(TIF)

Figure S3 **Cartoon illustrating the scheme used to acquire optical sections through unfed and fed nymphal midguts isolated at 48 and 72 h post-placement.** Serial Z-series confocal images were generated by obtaining 1-µm optical sections through the depth of unfed and fed midguts as described in Materials and Methods. (A) Cartoon depicting the scheme used to image individual diverticulum from unfed and 48 h-fed nymphal midguts; for these types of specimens, optical sections were acquired in the coronal plane through the full thickness of a midgut. (B) Cartoon depicting the scheme used to acquire images of an individual diverticulum from a 72 h-fed midgut in the coronal and transverse planes. (C) Zoom-in of coronal views for 72 h-fed nymphal midguts infected with either WT or *ΔrpoS* organisms illustrating the depths at which fluorescent spirochetes can be visualized by confocal microscopy. (D) Zoom-in of 72 h-fed nymphal midguts infected with *ΔrpoS* organisms imaged in the transverse plane after a portion of the blood meal had been removed; note the difference in the luminal space in the coronal and transverse planes. Solid-line arrows are used to indicate the laser beam, while a dashed line and “eye” are used to represent what is detected by the microscope's photomultiplier tube. Abbreviations: LS, luminal surface; MW, midway through the epithelial layer; and BM, basement membrane.
(TIF)

Figure S4 **Spirochetes introduced by immersion undergo biphasic dissemination.** (A) Spirochete burdens are identical in unfed and fed nymphs that acquired WT *Bb* as larvae by immersion or naturally by feeding on infected mice. Burdens were determined by qPCR; values represent the means ± SDs from three independent experiments. (B) The distribution of WT-*gfp* spirochetes in nymphs infected as larvae by immersion is highly similar to that of spirochetes within naturally-infected nymphs [30]. Composite images depicting midguts of nymphs infected by immersion are the same as those presented in Figures 2 and 3, while the images of midguts isolated from naturally-infected nymphs were taken from [30] and used with permission. The leftmost images in each panel “Full Composite” depict the full thickness of the midgut, while 3-µm composite images show spirochetes at the luminal surface, midway through the epithelial layer, and at the basement membrane. A detailed schematic indicating how confocal images of unfed and fed midguts were acquired is presented in Figure S3. Green represents GFP^+^ spirochetes and red indicates midgut epithelial cells labeled with FM4-64; scale bars = 25 µm. Images of naturally infected nymphs are reproduced with permission from Live imaging reveals a biphasic mode of dissemination of *Borrelia burgdorferi* within ticks; published in Volume 119, Issue 12 (December 1,2009), *J Clin Invest*. 2009; 119(12):3652–3665. doi10.117/JCI39401. Copyright © 2009, American Society for Clinical Investigation [30].
(TIF)

Figure S5 **Complementation of Δ** ***rpoS Bb*** **with a wild-type copy of** ***rpoS*** **restores expression of RpoS-dependent genes following temperature-shift.** Whole cell *B. burgdorferi* lysates were prepared from WT (Bb914), Δ*rpoS* (Bb1058) and RpoS-complemented (SE186) isolates, separated by SDS-PAGE and stained with silver as described in Materials and Methods.
(TIF)

Figure S6 ***ΔrpoS*** **organisms have an altered morphology and distribution pattern within 72 h-fed midguts.** Representative composite images of cryosectioned midguts infected with (A) WT-*gfp* or (B) *ΔrpoS-gfp Bb*; green represents spirochetes expressing GFP while red indicates midgut epithelial cells labeled with FM4-64; scale bars = 25 µm.
(TIF)

Figure S7 **Loss of RpoS and CoADR enhances round body formation under nutrient-limiting conditions** ***in vitro*** **.** Representative images of (A) WT-*gfp*, (B) *ΔrpoS-gfp*, (C) complemented *ΔrpoS-gfp*, (D) *Δcdr*, and (E) complemented *Δcdr* isolates after 3 days in RPMI; scale bar = 50 µm.
(TIF)

Table S1 **Oligonucleotides used in this study.**
(DOC)

Table S2 **Virulence of** ***B. burgdorferi*** **strains following needle-inoculation.**
(DOC)

Table S3 **Known and/or putative RpoS-dependent tick midgut adhesins.**
(DOC)

Video S1 **Round bodies generated** ***in vitro*** **rapidly elongate into spirochetes.** Spirochetes were transformed into round bodies by culturing either WT-*gfp* or *ΔrpoS-gfp* isolates in RPMI for 4 days. The video is arranged into two segments depicting the rapid transformation of WT-*gfp* or *ΔrpoS-gfp* round bodies into normal spirochetes after the addition of BSK-II (see the upper left hand corner for the time stamp).
(MOV)
