# Supplementary figures and images for: Borrelia burgdorferi Requires the Alternative Sigma Factor RpoS for Dissemination within the Vector during Tick-to-Mammal Transmission
Source: PLoS Pathog. 2012 Feb 16;8(2):e1002532. doi: 10.1371/journal.ppat.1002532 (PMC3280991; doi:10.1371/journal.ppat.1002532)

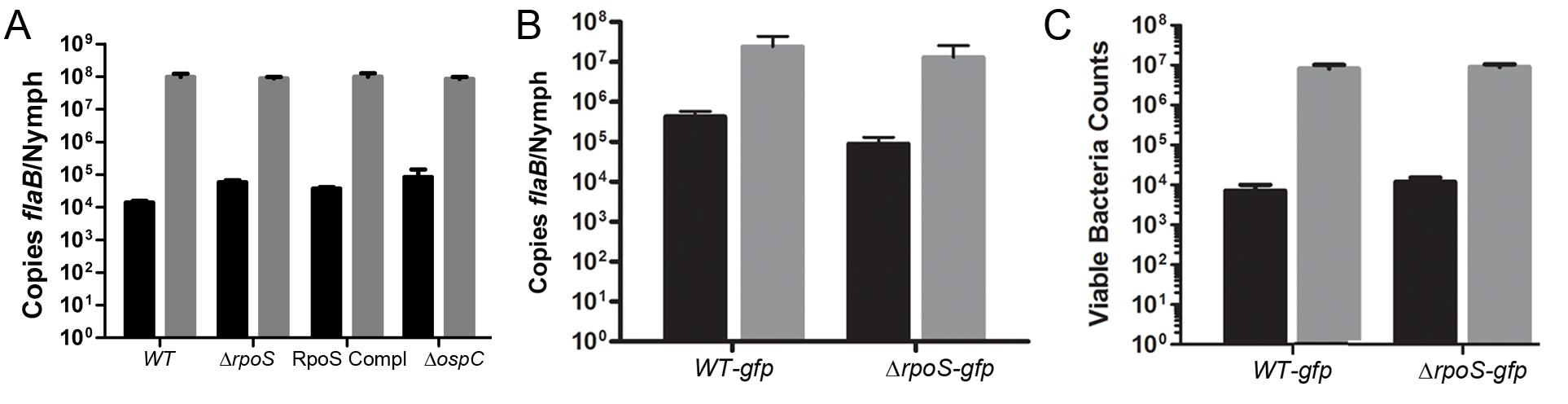

Supplement: Figure S1 — Spirochete persistence and survival in I. scapularis are not affected by loss of either RpoS or OspC. (A) Spirochete burdens of WT, ΔrpoS, RpoS complemented RpoS Compl) and ΔospC before and after feeding on naïve C3H/HeJ mice. The nymphs used in these experiments were used in Table 2. WT-gfp and ΔrpoS-gfp (B) burdens determined by qPCR and (C) viability, assessed by semi-solid plating, are highly similar in unfed (black) and fed (gray) nymphs. Values represent the means ± SEMs from three independent experiments. (TIF) [file ppat.1002532.s001.tif]

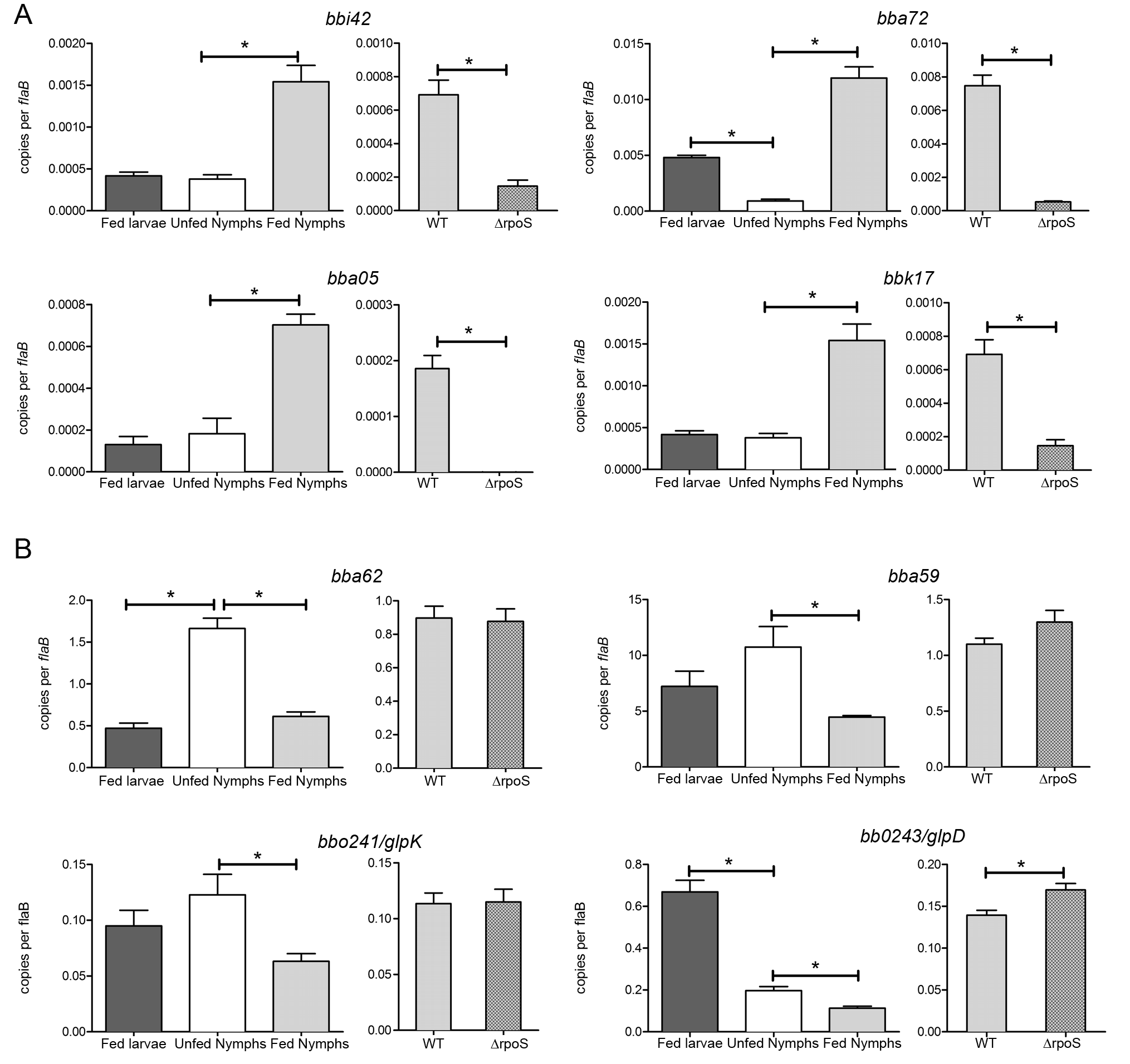

Supplement: Figure S2 — Contours of the RpoSBb regulon in I. scapularis . qRT-PCR analysis of (A) absolutely and (B) partially RpoS-dependent upregulated genes selected from microarray data derived from Bb cultivated within DMCs [13]. Expression profiling was performed using fed larvae, unfed and fed nymphs that had been naturally-infected with WT Bb as well as fed nymphs that had been infected as larvae by immersion with either WT-gfp or ΔrpoS-gfp isolates. Values represent the average flaB-normalized transcript copy number ± SEM for each gene; average values are considered significantly different when p is ≤0.05 (indicated by asterisks). (TIF) [file ppat.1002532.s002.tif]

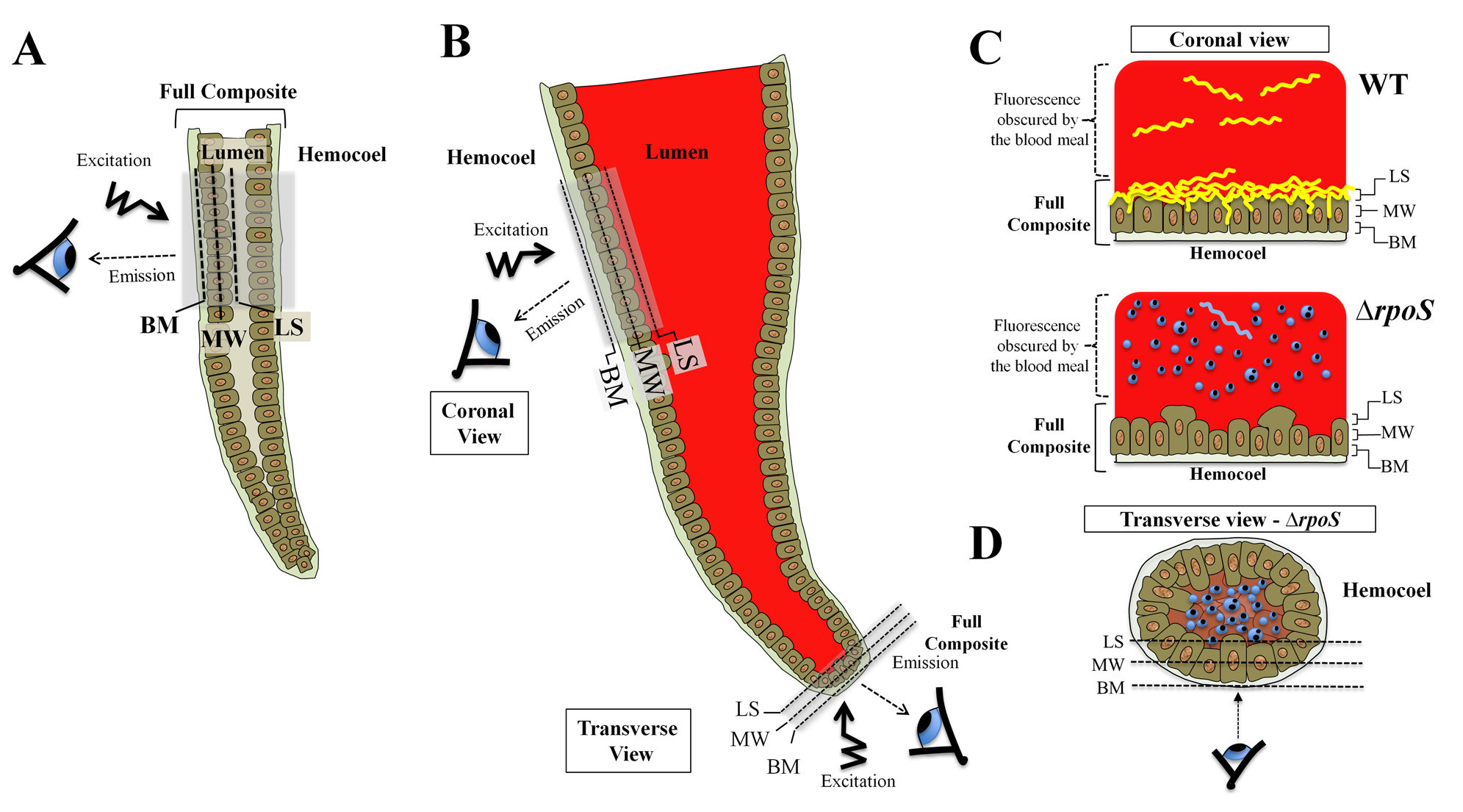

Supplement: Figure S3 — Cartoon illustrating the scheme used to acquire optical sections through unfed and fed nymphal midguts isolated at 48 and 72 h post-placement. Serial Z-series confocal images were generated by obtaining 1-µm optical sections through the depth of unfed and fed midguts as described in Materials and Methods. (A) Cartoon depicting the scheme used to image individual diverticulum from unfed and 48 h-fed nymphal midguts; for these types of specimens, optical sections were acquired in the coronal plane through the full thickness of a midgut. (B) Cartoon depicting the scheme used to acquire images of an individual diverticulum from a 72 h-fed midgut in the coronal and transverse planes. (C) Zoom-in of coronal views for 72 h-fed nymphal midguts infected with either WT or ΔrpoS organisms illustrating the depths at which fluorescent spirochetes can be visualized by confocal microscopy. (D) Zoom-in of 72 h-fed nymphal midguts infected with ΔrpoS organisms imaged in the transverse plane after a portion of the blood meal had been removed; note the difference in the luminal space in the coronal and transverse planes. Solid-line arrows are used to indicate the laser beam, while a dashed line and “eye” are used to represent what is detected by the microscope's photomultiplier tube. Abbreviations: LS, luminal surface; MW, midway through the epithelial layer; and BM, basement membrane. (TIF) [file ppat.1002532.s003.tif]

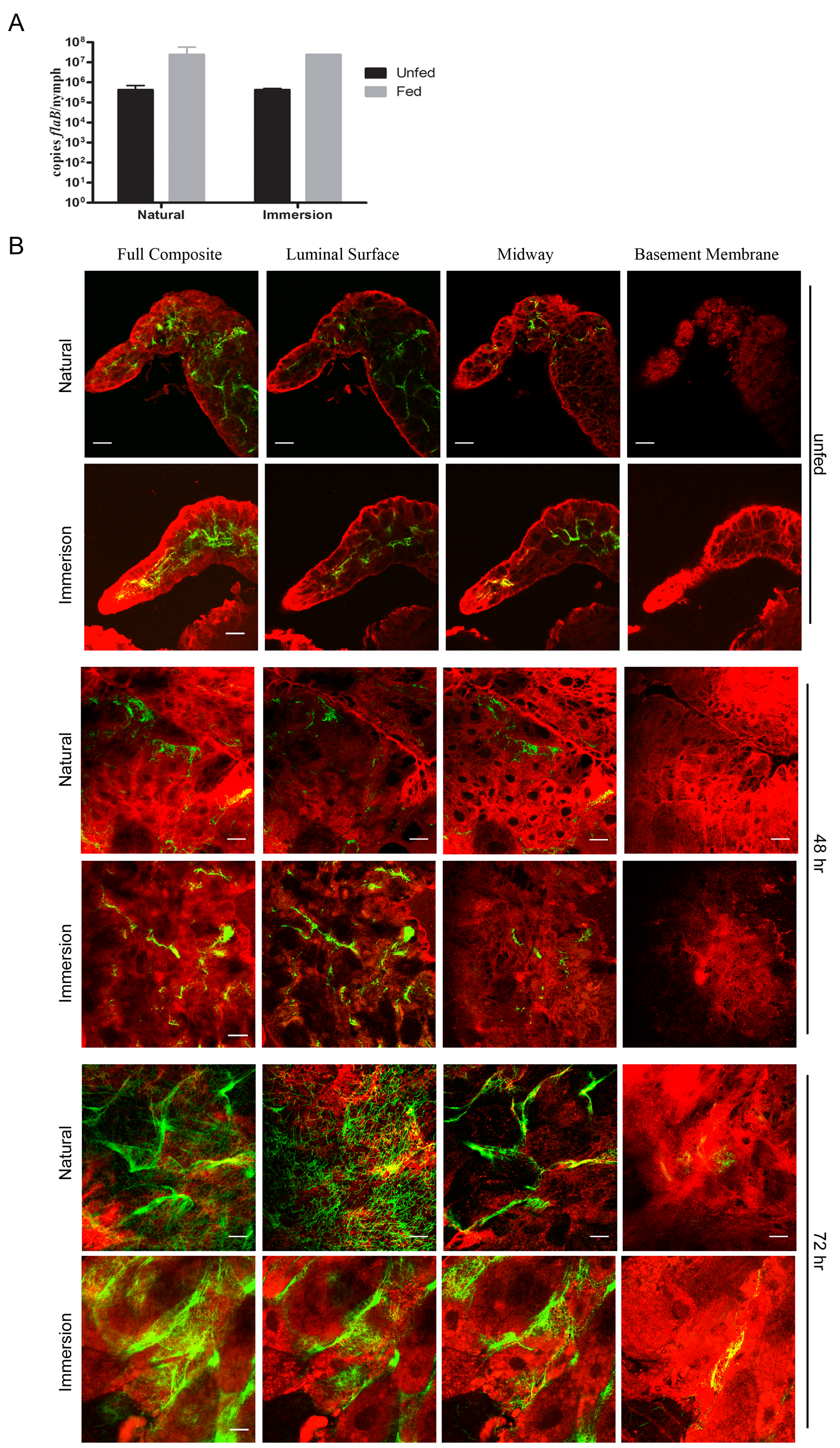

Supplement: Figure S4 — Spirochetes introduced by immersion undergo biphasic dissemination. (A) Spirochete burdens are identical in unfed and fed nymphs that acquired WT Bb as larvae by immersion or naturally by feeding on infected mice. Burdens were determined by qPCR; values represent the means ± SDs from three independent experiments. (B) The distribution of WT-gfp spirochetes in nymphs infected as larvae by immersion is highly similar to that of spirochetes within naturally-infected nymphs [30]. Composite images depicting midguts of nymphs infected by immersion are the same as those presented in Figures 2 and 3, while the images of midguts isolated from naturally-infected nymphs were taken from [30] and used with permission. The leftmost images in each panel “Full Composite” depict the full thickness of the midgut, while 3-µm composite images show spirochetes at the luminal surface, midway through the epithelial layer, and at the basement membrane. A detailed schematic indicating how confocal images of unfed and fed midguts were acquired is presented in Figure S3. Green represents GFP+ spirochetes and red indicates midgut epithelial cells labeled with FM4-64; scale bars = 25 µm. Images of naturally infected nymphs are reproduced with permission from Live imaging reveals a biphasic mode of dissemination of Borrelia burgdorferi within ticks; published in Volume 119, Issue 12 (December 1,2009), J Clin Invest. 2009; 119(12):3652–3665. doi10.117/JCI39401. Copyright © 2009, American Society for Clinical Investigation [30]. (TIF) [file ppat.1002532.s004.tif]

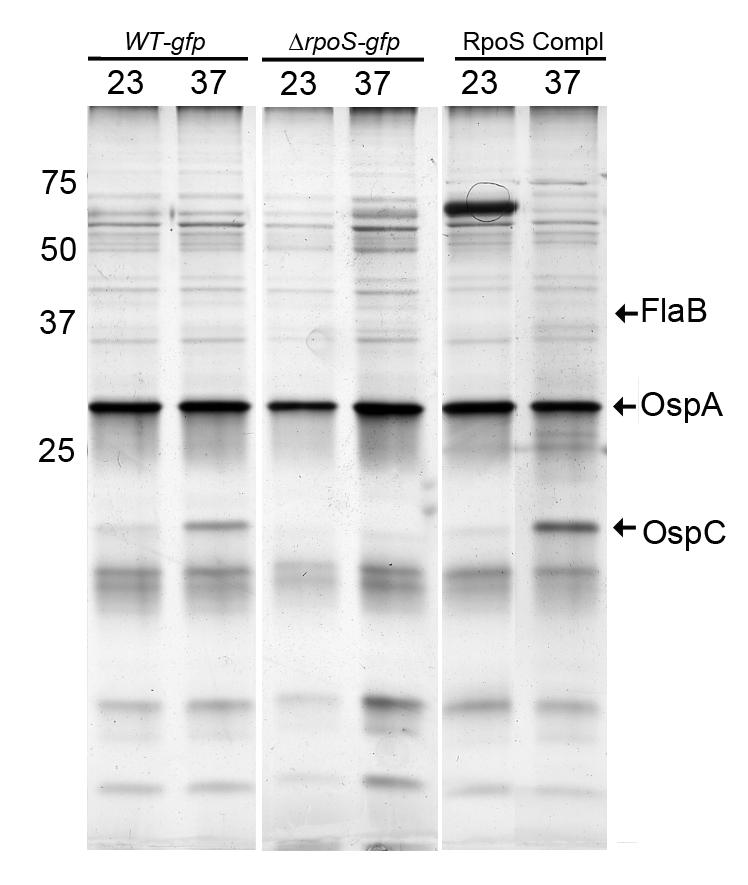

Supplement: Figure S5 — Complementation of Δ rpoS Bb with a wild-type copy of rpoS restores expression of RpoS-dependent genes following temperature-shift. Whole cell B. burgdorferi lysates were prepared from WT (Bb914), ΔrpoS (Bb1058) and RpoS-complemented (SE186) isolates, separated by SDS-PAGE and stained with silver as described in Materials and Methods. (TIF) [file ppat.1002532.s005.tif]

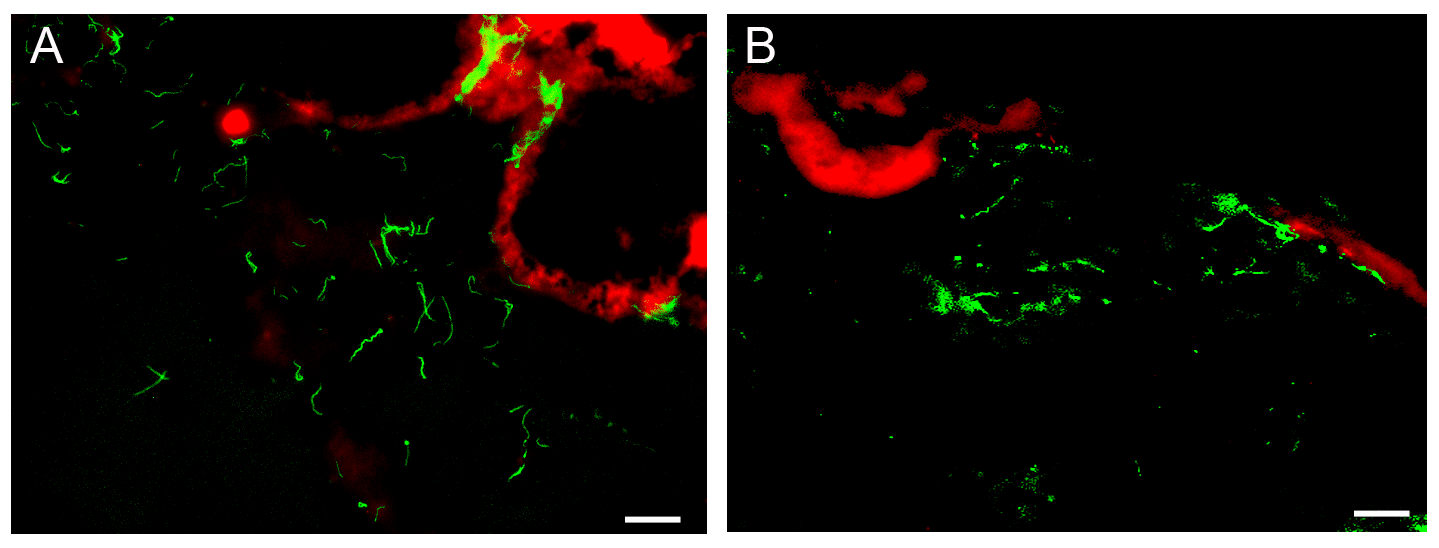

Supplement: Figure S6 — ΔrpoS organisms have an altered morphology and distribution pattern within 72 h-fed midguts. Representative composite images of cryosectioned midguts infected with (A) WT-gfp or (B) ΔrpoS-gfp Bb; green represents spirochetes expressing GFP while red indicates midgut epithelial cells labeled with FM4-64; scale bars = 25 µm. (TIF) [file ppat.1002532.s006.tif]

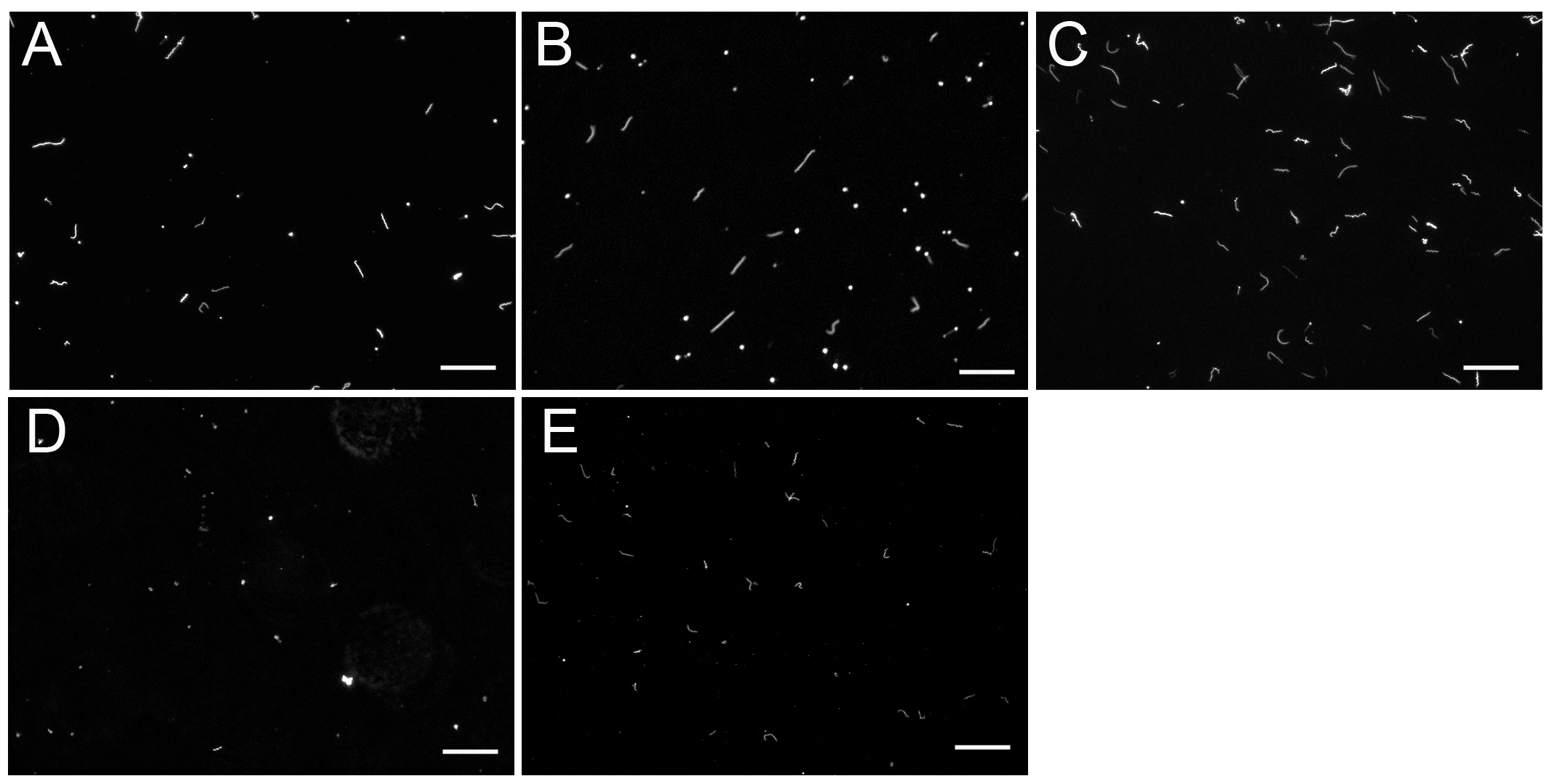

Supplement: Figure S7 — Loss of RpoS and CoADR enhances round body formation under nutrient-limiting conditions in vitro . Representative images of (A) WT-gfp, (B) ΔrpoS-gfp, (C) complemented ΔrpoS-gfp, (D) Δcdr, and (E) complemented Δcdr isolates after 3 days in RPMI; scale bar = 50 µm. (TIF) [file ppat.1002532.s007.tif]
