# Supplementary material for: Borrelia burgdorferi Requires the Alternative Sigma Factor RpoS for Dissemination within the Vector during Tick-to-Mammal Transmission
Source: PLoS Pathog. 2012 Feb 16;8(2):e1002532. doi: 10.1371/journal.ppat.1002532 (PMC3280991; doi:10.1371/journal.ppat.1002532)
Supplement: Table S1 — Oligonucleotides used in this study. (DOC) [file ppat.1002532.s008.doc]

**Table S1. Oligonucleotides used in this study**

| **Prime**r | **Sequence (5’-3’** | **Purpose** | **Reference** |
| --- | --- | --- | --- |
| PrpoS-F' | GACTGCAGAACAAATCTTAAAAATAAAGAGGG | Confirmation of *rpoS* mutation | [1] |
| rpoS-R | CTTGCAAATGCCTGAGTTATTGCA | Confirmation of *rpoS* mutation | [1] |
| rpoS-prom(extd) | GAGCATGCAACAAATCTTAAAAATAAAGAGGG | Generation of RpoS complement | [1] |
| rpoS-R’XbaI | ACTCTAGATTAATTTATTTCTTCTTTTAATTTTTA | Generation of RpoS complement | [1] |
| flaB-R | CTTTTCTCTGGTGAGGGAGCTC | qPCR and qRT-PCR | [2] |
| flaB-F | GCTCCTTCCTGTTGAACACCC | qPCR and qRT-PCR | [2] |
| flaB-Taqman Probe | FAM-CTTGAACCGGTGCAGCCTGAGCA-BHQ1 | qPCR and qRT-PCR | [2] |
| PflgB(NgoMIV) | GCCGGCTAATACCCGAGCTTGAAGGAG | Construction of Δ*ospC* mutant | [3] |
| KanR(BamH1) | GGCGAATTACCTAGGGCCGTCCC | Construction of Δ*ospC* mutant | [3] |
| BBB18#1F | CGCCTACAGATTTGACAGG | Construction of Δ*ospC* mutant | This study |
| BBB19#1R(NgoMIV) | CTGTAAGATTGCCGGCTTTAACAGACTCATCAGC | Construction of Δ*ospC* mutant | This study |
| BBB19#1F(NgoMIV) | GCTGATGAGTCTGTTAAAGCCGGCAATCTTACAG | Construction of Δ*ospC* mutant | This study |
| BBB22#1R | GGACTTTCTGCCACAACAGGGGC | Construction of Δ*ospC* mutant | This study |
| bbb19/ospC-F | AGGGAAAGGTGGGAATACATC | qRT-PCR | [1] |
| bbb19/ospC-R | TGTTCCATTATGCCCCGC | qRT-PCR | [1] |
| bba25/dbpA-F | GGGTAGTGGGGTATCAGAAAATC | qRT-PCR | [1] |
| bba25/dbpA-R | GAGCTGTAGTTGGAGGATTCTC | qRT-PCR | [1] |
| bb0680/mcp4-F | GGTCTAAACAAAGCGAAAAAAAGG | qRT-PCR | [1] |
| bb0680/mcp4-R | GAATCTAAATCTATCAAAACTATCTGCCAC | qRT-PCR | [1] |
| bba07-F | TAGCAATCCCGACAAGTTTAAT | qRT-PCR | This study |
| bba07-R | AGAGCCATTTTAGCCTTTCTTTT | qRT-PCR | This study |
| bbi42-F | GTTGATAAGCAGTGGGTCTAG | qRT-PCR | This study |
| bbi42-R | GTACTCCCAGTGAGTGACTA | qRT-PCR | This study |
| bba72-F | GCATAAGGAGAGTGTTTTGAC | qRT-PCR | This study |
| bba72-R | TTTATATCGGTGCGGCTTT | qRT-PCR | This study |
| bba05-F | TATTGGCAAGTCAAGATAC | qRT-PCR | This study |
| bba05-R | TTACTAACACCTCATCATTG | qRT-PCR | This study |
| bbk17-F | GTTCTTTTCTTGTTCCATCAAACTT | qRT-PCR | [4] |
| bbk17-R | ATGCCATCAATACCATTAACATTG | qRT-PCR | [4] |
| bb0728/cdr-F | GACGCTGTTATACTTGCTACCG | qRT-PCR | [4] |
| bb0728/cdr-R | GAAGCTGAGCCCAATGTGCCT | qRT-PCR | [4] |
| bb0670/cheW3-F | TTGATACTGATTACTTGCCTTG | qRT-PCR | This study |
| bb0670/cheW3-R | TCTCCTTTCCACTACCACAAC | qRT-PCR | This study |
| bba15/ospA-F | GTTTTGTAATTTCAACTGCTGACC | qRT-PCR | [4] |
| bba15/ospA-R | CTGCAGCTTGGAATTCAGGCACTT | qRT-PCR | [4] |
| bb0240/glpF-F | AAGTCCCGAATACCAGGAGAAAT | qRT-PCR | This study |
| bb0240/glpF-R | TTCTTGCTGCTGTGTAAATACCAAA | qRT-PCR | This study |
| bb0365-F | TTCACGCTATGGGAGTAGTTC | qRT-PCR | This study |
| bb0365-R | AGGAAGGTCTTGGCATCTGA | qRT-PCR | This study |
| bba52-F | TTGGTCGTGGGATTTTAATAGATTCTA | qRT-PCR | This study |
| bba52-R | TGAGGCTTTTGATTGTGGGTTT | qRT-PCR | This study |
| bba62-F | GTTGCTTGCGAAACTACAAGA | qRT-PCR | [4] |
| bba62-R | CATTGACTTTGTCATAGGTTGCTT | qRT-PCR | [4] |
| bba59-F | TCAAAAAACTCAAGACCTTCCAAAA | qRT-PCR | This study |
| bba59-R | AATTCACTTTCTGCACCGTTAAGAT | qRT-PCR | This study |
| bb0241/glpK-F | TGAAATTGACGCTATTGGAA | qRT-PCR | This study |
| bb0241/glpK-R | CATTGTAGATGGGCTTTCCT | qRT-PCR | This study |
| bb0243/glpD-F | TTGTGGAAGCACTGACATTCC | qRT-PCR | This study |
| bb0243/glpD-R | AAGGTAGCCGTGCAACTTTAA | qRT-PCR | This study |

References Cited:

1. Caimano MJ, Eggers CH, Hazlett KR, Radolf JD (2004) RpoS is not central to the general stress response in *Borrelia burgdorferi* but does control expression of one or more essential virulence determinants. Infection and Immunity 72: 6433-6445.

2. Yang XF, Pal U, Alani SM, Fikrig E, Norgard MV (2004) Essential role for OspA/B in the life cycle of the Lyme disease spirochete. Journal of Experimental Medicine 199: 641-648.

3. Bono JL, Elias AF, Kupko JJ, Stevenson B, Tilly K, et al. (2000) Efficient targeted mutagenesis in *Borrelia burgdorferi* Journal of Bacteriology 182: 2445-2452.

4. Caimano MJ, Iyer R, Eggers CH, Gonzalez C, Morton EA, et al. (2007) Analysis of the RpoS regulon in *Borrelia burgdorferi* in response to mammalian host signals provides insight into RpoS function during the enzootic cycle. Molecular Microbiology 65: 1193-1217.
