# Supplementary material for: Borrelia burgdorferi Requires the Alternative Sigma Factor RpoS for Dissemination within the Vector during Tick-to-Mammal Transmission
Source: PLoS Pathog. 2012 Feb 16;8(2):e1002532. doi: 10.1371/journal.ppat.1002532 (PMC3280991; doi:10.1371/journal.ppat.1002532)
Supplement: Table S2 — Virulence of B. burgdorferi strains following needle-inoculation. (DOC) [file ppat.1002532.s009.doc]

**Table S2. Virulence of *B. burgdorferi* strains following needle-inoculation**

| **Strain^a^** | **Number of Positive Cultures^b,c^** | | | |
| --- | --- | --- | --- | --- |
|  | **Ear** | **Joint** | **Bladder** | **Heart** |
| **CE162** | 5/5 | 4/5 | 5/5 | 4/5 |
| **Bb914** | 5/5 | 5/5 | 5/5 | 4/5 |
| **CE303** | 0/5 | 0/5 | 0/5 | 0/5 |
| **CE174** | 0/5 | 0/5 | 0/5 | 0/5 |
| **Bb1058** | 0/5 | 0/5 | 0/5 | 0/5 |
| **SE168** | 5/5 | 5/5 | 5/5 | 4/5 |
| **SE186** | 5/5 | 5/5 | 5/5 | 5/5 |

^a^ C3H/HeJ mice (5 per isolate) were inoculated intradermally with 1 x 10^4^ spirochetes

^b^ Mice were sacrificed 4 weeks post-inoculation and the indicated sites were cultured in BSK-II medium.

^c^ Cultures were monitored for the presence of spirochetes by dark field microscopy for up to 8 weeks.
