# Supplementary material for: Borrelia burgdorferi Requires the Alternative Sigma Factor RpoS for Dissemination within the Vector during Tick-to-Mammal Transmission
Source: PLoS Pathog. 2012 Feb 16;8(2):e1002532. doi: 10.1371/journal.ppat.1002532 (PMC3280991; doi:10.1371/journal.ppat.1002532)
Supplement: Table S3 — Known and/or putative RpoS-dependent tick midgut adhesins. (DOC) [file ppat.1002532.s010.doc]

**Table S3. Known and/or putative RpoS-dependent tick midgut** adhesins

| **Gene^a,b^** | **Description** | **Classification^c^** | **RpoS Regulation^d^** |
| --- | --- | --- | --- |
| **bb0384** | Basic membrane protein C (bmpC) | LP | Upregulated |
| **bba04** | S2 antigen | LP | Upregulated |
| **bba05** | S1 antigen | LP | Upregulated |
| **bba07** | chpAI protein | LP | Upregulated |
| **bba24** | Decorin binding protein A (dbpA) | LP | Upregulated |
| **bba25** | Decorin binding protein B (dbpB) | LP | Upregulated |
| **bba32** | hypothetical protein | LP | Upregulated |
| **bba33** | hypothetical protein | LP | Upregulated |
| **bba36** | Lipoprotein | LP | Upregulated |
| **bba57** | Hypothetical protein | LP | Upregulated |
| **bba65** | Hypothetical protein | LP | Upregulated |
| **bba66** | P35 antigen | LP | Upregulated |
| **bba72** | Hypothetical protein | LP | Upregulated |
| **bba73** | P35 antigen | LP | Upregulated |
| **bbb09** | Hypothetical protein | LP | Upregulated |
| **bbb14** | Hypothetical protein | LP | Upregulated |
| **bbd001** | Hypothetical protein | LP | Upregulated |
| **bbi42** | Outer membrane protein | LP | Upregulated |
| **bbo26** | Hypothetical protein | LP | Upregulated |
| **bbp27** | Rev | LP | Upregulated |
| **bb0400** | Hypothetical protein | OMP | Upregulated |
| **bb0563** | Hypothetical protein | OMP | Upregulated |
| **bbg23** | Hypothetical protein | OMP | Upregulated |
| **bbg24** | Hypothetical protein | OMP | Upregulated |
| **bbj23** | Hypothetical protein | OMP | Upregulated |
| **bbl01** | Hypothetical protein | OMP | Upregulated |
| **bbl03** | Hypothetical protein | OMP | Upregulated |
| **bb0365** | Lipoprotein LA7 | LP | Repressed |
| **bba15** | Outer surface protein A (ospA) | LP | Repressed |
| **bba16** | Outer surface protein B (ospB) | LP | Repressed |
| **bba69** | Hypothetical protein | LP | Repressed |
| **bbf20** | Hypothetical protein | LP | Repressed |
| **bbg01** | Hypothetical protein | LP | Repressed |
| **bbi29** | Hypothetical protein | LP | Repressed |
| **bbi38** | Hypothetical protein | LP | Repressed |
| **bbi39** | Hypothetical protein | LP | repressed |
| **bbk01** | Hypothetical protein | LP | repressed |
| **bbk19** | Hypothetical protein | LP | repressed |

^a^Gene designation based on *Borrelia burgdorferi* B31 genome page at the J. Craig Venter Institute website (http://cmr.jcvi.org/tigr-scripts/CMR/GenomePage.cgi?org=gbb)

^b^*bba64* has been removed from the table because a phenotype was not detected within ticks [1,2].

^c^Classification as a lipoprotein (LP) and outer membrane protein (OMP) determined as described in materials and methods using the algorithms developed by Cox *et al*.[3] and Setubal *et al.*[4].

^d^Regulation based on microarray analyses performed by [5]

**References:**

1. Patton TG, Dietrich G, Dolan MC, Piesman J, Carroll JA, et al. (2011) Functional analysis of the *Borrelia burgdorferi* *bba64* gene product in murine infection via tick infestation. PLoS One 6: e19536.

2. Gilmore RD, Jr., Howison RR, Dietrich G, Patton TG, Clifton DR, et al. (2010) The *bba64* gene of *Borrelia burgdorferi*, the Lyme disease agent, is critical for mammalian infection via tick bite transmission. Proc Natl Acad Sci U S A 107: 7515-7520.

3. Cox DL, Luthra A, Dunham-Ems S, Desrosiers DC, Salazar JC, et al. (2010) Surface immunolabeling and consensus computational framework to identify candidate rare outer membrane proteins of Treponema pallidum. Infect Immun 78: 5178-5194.

4. Setubal JC, Reis M, Matsunaga J, Haake DA (2006) Lipoprotein computational prediction in spirochaetal genomes. Microbiology 152: 113-121.

5. Caimano MJ, Iyer R, Eggers CH, Gonzalez C, Morton EA, et al. (2007) Analysis of the RpoS regulon in *Borrelia burgdorferi* in response to mammalian host signals provides insight into RpoS function during the enzootic cycle. Molecular Microbiology 65: 1193-1217.
